# How many biological replicates are needed in an RNA-seq experiment and which differential expression tool should you use?

**DOI:** 10.1261/rna.053959.115

**Published:** 2016-06

**Authors:** Nicholas J. Schurch, Pietá Schofield, Marek Gierliński, Christian Cole, Alexander Sherstnev, Vijender Singh, Nicola Wrobel, Karim Gharbi, Gordon G. Simpson, Tom Owen-Hughes, Mark Blaxter, Geoffrey J. Barton

**Affiliations:** 1 Division of Computational Biology, College of Life Sciences, University of Dundee, Dundee DD1 5EH, United Kingdom; 2 Division of Gene Regulation and Expression, College of Life Sciences, University of Dundee, Dundee DD1 5EH, United Kingdom; 3 Edinburgh Genomics, University of Edinburgh, Edinburgh EH9 3JT, United Kingdom; 4 Division of Plant Sciences, College of Life Sciences, University of Dundee, Dundee DD1 5EH, United Kingdom; 5 Division of Biological Chemistry and Drug Discovery, College of Life Sciences, University of Dundee, Dundee DD1 5EH, United Kingdom

**Keywords:** RNA-seq, benchmarking, differential expression, replication, yeast, experimental design, statistical power

## Abstract

RNA-seq is now the technology of choice for genome-wide differential gene expression experiments, but it is not clear how many biological replicates are needed to ensure valid biological interpretation of the results or which statistical tools are best for analyzing the data. An RNA-seq experiment with 48 biological replicates in each of two conditions was performed to answer these questions and provide guidelines for experimental design. With three biological replicates, nine of the 11 tools evaluated found only 20%–40% of the significantly differentially expressed (SDE) genes identified with the full set of 42 clean replicates. This rises to >85% for the subset of SDE genes changing in expression by more than fourfold. To achieve >85% for all SDE genes regardless of fold change requires more than 20 biological replicates. The same nine tools successfully control their false discovery rate at ≲5% for all numbers of replicates, while the remaining two tools fail to control their FDR adequately, particularly for low numbers of replicates. For future RNA-seq experiments, these results suggest that at least six biological replicates should be used, rising to at least 12 when it is important to identify SDE genes for all fold changes. If fewer than 12 replicates are used, a superior combination of true positive and false positive performances makes *edgeR* and *DESeq2* the leading tools. For higher replicate numbers, minimizing false positives is more important and *DESeq* marginally outperforms the other tools.

## INTRODUCTION

RNA-seq has now supplanted microarrays as the technology of choice for genome-wide differential gene expression (DGE) experiments. In any experimental design, selecting the appropriate number of biological replicates is a trade-off between cost and precision. For microarray methods it has been shown that low replicate experiments often have insufficient statistical power to call DGE correctly (Pan et al. 2002) and cannot accurately measure the natural biological variability (Churchill 2002). Although it is widely appreciated that increasing the number of replicates in an RNA-seq experiment usually leads to more robust results (Auer and Doerge 2010; Hansen et al. 2011; Busby et al. 2013; Liu et al. 2014), the precise relationship between replicate number and the ability to correctly identify the differentially expressed genes (i.e., the statistical power of the experiment) has not been fully explored.

The rise of RNA-seq technology has led to the development of many tools for analyzing DGE from these data (e.g., Anders and Huber 2010; Hardcastle and Kelly 2010; Robinson et al. 2010; Wang et al. 2010; Tarazona et al. 2011; Li et al. 2012; Lund et al. 2012; Trapnell et al. 2012; Leng et al. 2013; Li and Tibshirani 2013; Frazee et al. 2014; Law et al. 2014; Love et al. 2014; Moulos and Hatzis 2015). Each tool makes assumptions about the statistical properties inherent to RNA-seq data and they exploit a range of normalization and analysis techniques to compute the magnitude of a DGE result and estimate its significance. Several studies have generated data specifically for the purpose of testing the assumptions intrinsic to DGE methods (Marioni et al. 2008; SEQC/MAQC-III Consortium 2014), but most rely either on RNA-seq data sets designed to test biological hypotheses (Bullard et al. 2010; Rapaport et al. 2013; Seyednasrollah et al. 2013) or simulated data (Busby et al. 2013; Soneson 2014), or a combination of the two (Kvam et al. 2012; Li et al. 2012; Dillies et al. 2013; Guo et al. 2013; Soneson and Delorenzi 2013; Burden et al. 2014). The majority of studies based on analysis of experimental RNA-seq data rely on data from experiments with fewer than five replicates per condition (Marioni et al. 2008; Bullard et al. 2010; Kvam et al. 2012; Li et al. 2012; Busby et al. 2013; Dillies et al. 2013; Rapaport et al. 2013; SEQC/MAQC-III Consortium 2014; Soneson 2014), limiting their ability to compare the performance of DGE tools as a function of replication.

Two studies explore higher replication by exploiting publicly available RNA-seq data from 21 individual clones of two laboratory strains of mouse (Bottomly et al. 2011; Soneson and Delorenzi 2013; Burden et al. 2014). Burden et al. (2014) consider false discovery rate (FDR) as the main metric for ranking five tools and conclude that at least six replicates per condition and multiplexing DGE tools gives the best results. Soneson and Delorenzi (2013) focus on the degree of concordance between tools as a metric for comparison and conclude that none of the 11 tools they tested perform well with fewer than three replicates. Nevertheless, since the experiments are from individual mice, the data may reflect interindividual variance in RNA expression as well as from other aspects of the experimental protocol. The same is true of studies in human that make use of data from individuals to explore higher sample replication in DGE (Guo et al. 2013; Seyednasrollah et al. 2013). Guo et al. (2013) expand the replicate number by comparing six tools using RNA-seq data from breast cancer tumor-normal paired samples from 53 individuals in The Cancer Genome Atlas (TCGA, The Cancer Genome Atlas Research Network 2008), using this primarily to guide the construction of a simulated data set. They conclude that all six of the tools they test suffer from oversensitivity but that *edgeR* represents the best compromise between accuracy and speed. Seyednasrollah et al. (2013) examine the performance of eight tools using mouse data (Bottomly et al. 2011) and lymphoblastoid cell data from a cohort of 56 unrelated Nigerian individuals from the HapMap project (The International HapMap Consortium 2005). They recommend *limma* and *DESeq* for data with fewer than five replicates per condition, finding that *edgeR* is “oversensitive” and suffers from high variability in its results while *SAMSeq* suffers from a lack of statistical power with few replicates. The idea of combining DGE methods is implemented in the novel tool *PANDORA*, which weights the results of different DGE tools according to their performance on test data and performs at least as well as the constituent tools (Moulos and Hatzis 2015).

In this paper, the performance of DGE tools is evaluated through the first highly replicated RNA-seq experiment designed specifically to test both the assumptions intrinsic to RNA-seq DGE tools (Gierliński et al. 2015) and to assess their performance. The paper focuses on 11 popular RNA-seq specific DGE tools (as judged by citations): *baySeq*, *cuffdiff*, *DEGSeq, DESeq*, *DESeq2*, *EBSeq*, *edgeR (exact* and *glm modes)*, *limma*, *NOISeq*, *PoissonSeq*, and *SAMSeq* (see Table 1 for references) and assesses their performance as a function of replicate number and fold change. The study provides general recommendations on:

How many replicates future RNA-seq experiments require to maximize the sensitivity and accuracy of DGE identification and quantification.
The most appropriate DGE tools to use to detect DE genes in RNA-seq experiments with a given number of replicates.

**TABLE 1.**
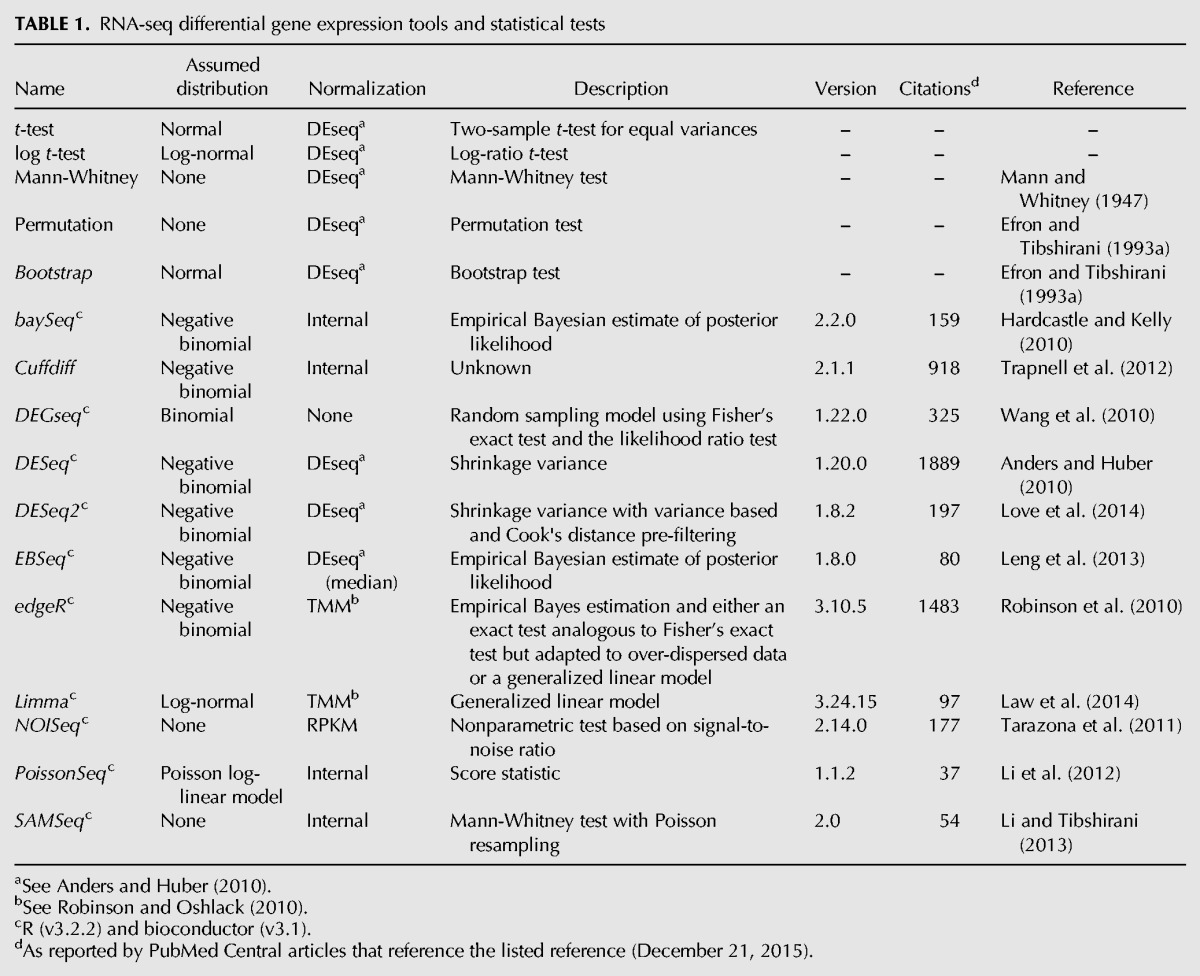
RNA-seq differential gene expression tools and statistical tests

## RESULTS

### Tool-specific gold standards

RNA was sequenced from 48 biological replicate samples of *Saccharomyces cerevisiae* in each of two well-studied experimental conditions; wild-type (WT) and a Δ*snf2* mutant. Quality control and data processing steps reject several replicates from each condition resulting in 42 WT and 44 Δ*snf2* biological replicates of “clean” data totaling ∼889M aligned reads (see Materials and Methods for a full description on the experiment, the mutant strain, the sequencing and the quality control and data processing steps). The data used for the performance comparison here represents a best-case scenario for the DGE tools since biological variation within conditions is low (Pearson's *R* > 0.97 for all pairs of replicates). In contrast, the mean Pearson's correlation (±1 SD) between replicates using the count data for four RNA-seq studies from the ReCount project (Frazee et al. 2011) show only $\bar{R}=0.86_{-0.09}^{+0.09}$ (Cheung et al. 2010), $\bar{R}=0.95_{-0.04}^{+0.04}$ (Bottomly et al. 2011), $\bar{R}=0.89_{-0.07}^{+0.07}$ (Montgomery et al. 2010; Pickrell et al. 2010), $\bar{R}=0.64_{-0.22}^{+0.22}$ (Wang et al. 2008).

The performance of each DGE tool as a function of replicate number and expression fold change was evaluated by comparing the DGE results from subsets of these replicates against the “gold standard” set of DGE results obtained for each tool with the full set of clean replicates. The tool-specific gold standards were computed by running the tool on the read-count-per-gene measurements from the full set of clean data and marking as “significantly differentially expressed” (SDE) those differentially expressed genes with multiple testing corrected *P*-values or FDRs ≤0.05. These gold-standard runs typically result in 60%–75% of the 7126 genes in the Ensembl v68 (Flicek et al. 2011) *S. cerevisiae* annotation being identified as SDE (except for *DEGSeq, NOIseq*, and *PoissonSeq*, which call >80% of the genes as SDE; see Supplemental Figs. S4, S10, S11A).

With the tool-specific gold standards defined, each DGE algorithm was run iteratively on *i* repeated subselections drawn from the set of clean replicates (without replacement). For each of the tools, bootstrap runs were performed with *i* = 100 iterations and *n*_*r*_ = 2,…,40 replicates in each condition (*cuffdiff* was significantly slower than the other tools so the number of iterations was reduced to *i* = 30 for this tool). For a given value of *n*_*r*_, the mean log_2_ transformed fold change [log_2_(*FC*)] and median adjusted *P*-value or FDR calculated across all the bootstrap iterations was considered representative of the measured behavior for each individual gene. Again, genes were marked as SDE when the adjusted *P*-value or FDR was ≤0.05. From these results, true positive, true negative, false positive, and false negative rates (hereafter TPR, TNR, FPR, FNR) were then calculated as a function of *n*_*r*_ for four arbitrary fold-change thresholds ($\left|\mathrm{lo}g_{2}(FC)\right|=T\in \left\{0,0.3,1,2\right\}$), by comparing the SDE genes from each bootstrap with the SDE genes from the tool's gold standard (see Materials and Methods for a detailed description of these calculations). Intrinsic to this method of measuring each tool's performance is the assumption that the large number of replicates in the full data set will enable each tool to unambiguously identify the “true” differentially expressed genes in the experiment.

### Tool performance

**FIGURE 1.**
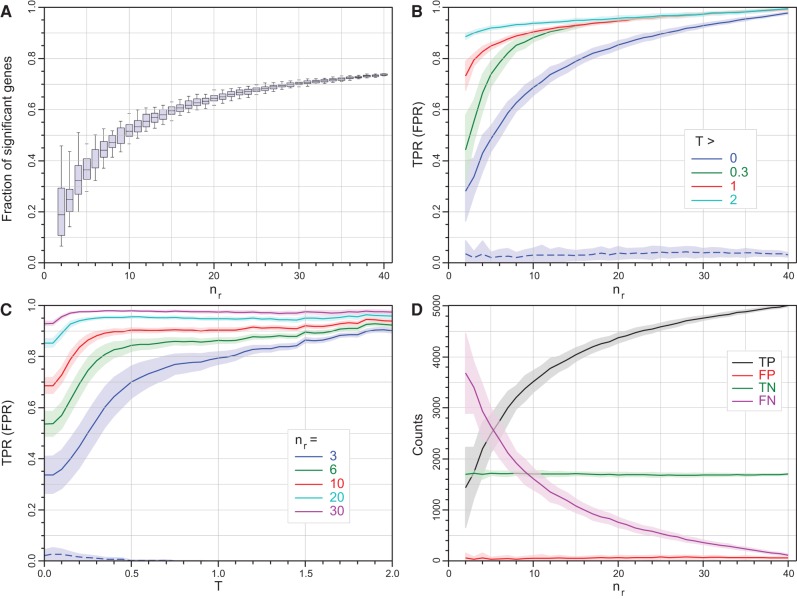
Statistical properties of *edgeR (exact)* as a function of $\left|\log_{2}(FC)\right|$ threshold, *T*, and the number of replicates, *n*_*r*_. Individual data points are not shown for clarity; however, the points comprising the lines are each an average over 100 bootstrap iterations, with the shaded regions showing the 1 SD limits. (*A*) The fraction of all (7126) genes called as SDE as a function of the number of replicates (boxplots show the median, quartiles and 95% limits across replicate selections within a bootstrap run). (*B*) Mean true positive rate (TPR) as a function of *n*_*r*_ for four thresholds *T*∈{0,0.3,1,2} (solid curves, the mean false positive rate [FPR] for *T* = 0 is shown as the dashed blue curve, for comparison). Data calculated for every Δ*n*_*r*_ = 1. (*C*) Mean TPR as a function of *T* for *n*_*r*_∈{3,6,10,20,30} (solid curves, again the mean FPR for *n*_*r*_ = 3 is shown as the dashed blue curve, for comparison). Data calculated every Δ*T* = 0.1. (*D*) The number of genes called as true/false positive/negative (TP, FP, TN, and FN) as a function of *n*_*r*_. The FPR remains extremely low with increasing *n*_*r*_ demonstrating that *edgeR* is excellent at controlling its false discovery rate. Data calculated for every Δ*n*_*r*_ = 1.

Figure 1 shows an example of the key performance data for *edgeR (exact)* (similar figures for *edgeR*'s generalized linear model mode and the other tools can be found in Supplemental Figs. S2–S12). The fraction of all genes *edgeR* (*exact*) calls as SDE increases as a function of *n*_*r*_ and the impact of sampling effects on this fraction shrinks as *n*_*r*_ increases (Fig. 1A). The TPR performance changes as a function of both replicate number and fold-change threshold (Fig. 1B,C). However, *edgeR (exact)* successfully controls its FDR for all combinations of both *n*_*r*_ and *T* and the primary effect of increasing the number of replicates or imposing a fold-change threshold is to increase the sensitivity of the tool, converting false negatives to true positives (Fig. 1D).

**FIGURE 2.**
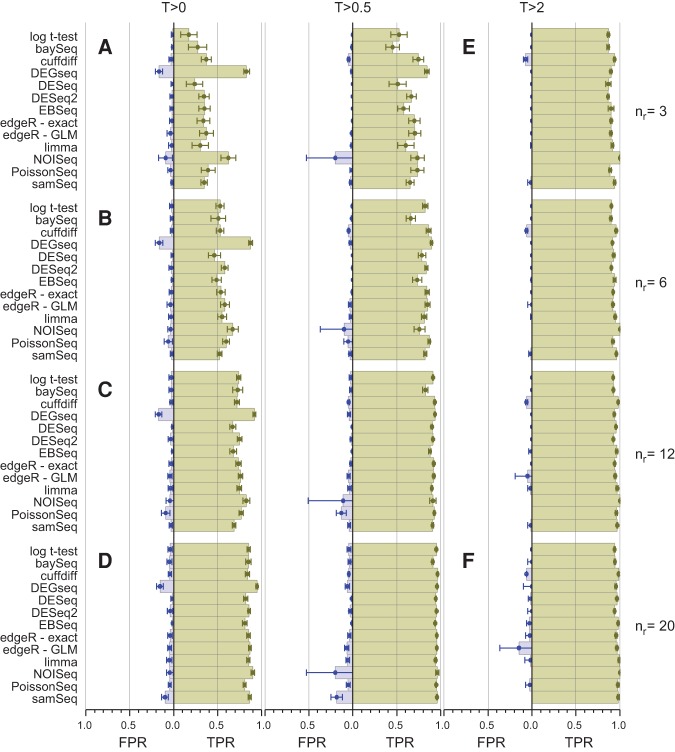
Comparison of the true positive rate (TPR) and false positive rate (FPR) performance for each of the DGE tools on low-, medium-, and highly replicated RNA-seq data (*n*_*r*_∈{3,6,12,20}—rows) for three |log_2_(*FC*)| thresholds (*T*∈{0,0.5,2}—columns). The TPRs and FPRs for each tool are calculated by comparing the mean number of true and false positives (TPs and FPs) calculated over 100 bootstrap iterations to the number of TPs and FPs calculated from the same tool using the full clean data set (error bars are 1 SD). Although the TPRs and FPRs from each tool are calculated by comparing each tool against itself rather than a tool-independent “gold standard” (albeit with the full clean data set), the results are comparable across tools except for *DEGSeq* which calls a significantly larger fraction of genes as DE for all values of T and *n*_*r*_ (Supplemental Fig. S4). In general, the TPR increases with increasing *n*_*r*_ (*A*–*D*) while both the TPR increases and the FPR decreases with increasing *T* (*A,D,E,F*). The TPR for bootstrap subselections with three replicates and no fold-change threshold is ∼20%–40% for all the tools except *NOISeq* and *DEGSeq* (*A*). For the highest fold-change genes (*T* = *2*), the tools show TPRs ≳85% and, with the exception of *cuffdiff* also show FPRs consistent with zero ([*E*] *NOISeq* and *PoissonSeq* produce no FPs for the highest threshold genes and thus no FPR is shown for them). For *T* = 2, increasing *n*_*r*_ provides only a modest increase in TPR (∼85% to ∼95%) irrespective of the tool (*E* and *F*). *PoissonSeq* and *BaySeq* show an increasing FPR with increasing *n*_*r*_ (*A*–*D*), and *cuffdiff* unexpectedly shows an increase in FPR with increasing *T*. *DESeq* appears more conservative than the other tools, consistently returning fewer FPs (particularly for high values of *n*_*r*_ [*D* and *F*]) and fewer TPs (particularly at low values of *n*_*r*_ [*A* and *E*]).

Figure 2 summarizes the performance of all 11 tools considered in this study as a function of replicate number and fold-change threshold. The TPR for bootstrap subselections with three replicates and no fold-change threshold (*n*_*r*_ = 3, *T* = 0) is ∼20%–40% for all the tools except *NOISeq* and *DEGSeq*, indicating that with this few replicates these experiments were unable to identify the majority of DE genes regardless of the tool used to analyze the data (Fig. 2A). *DEGSeq* and *NOISeq* both show strong TPR performance but this is coupled with high FPRs (*DEGSeq*: ∼17%, *NOISeq*: ∼9%). For *DEGSeq* in particular this originates from overestimating the number of SDE genes regardless of the number of replicates (Supplemental Fig. S4A). Excluding *DEGSeq*, the TPR performance for all the remaining tools is a strong function of fold change (Fig. 1C; Supplemental Figs. S2–S12C). For the highest fold-change genes (*T* = 2), these tools show TPRs ≳85% and with the exception of *cuffdiff* also show FPRs consistent with zero (Fig. 2E). These tools are successfully capturing the majority of the true differential expression signals for the most strongly changing genes from each tool's gold standard with as few as three replicates per condition. For this cohort of high fold-change SDE genes the TPR is largely insensitive to replicate number. Irrespective of the tool, increasing the number of replicates to *n*_*r*_ = 20 for *T* = 2 provides only a modest increase in TPR from ∼85% to ∼95% (Figs 1B, 2F; Supplemental Figs. S2–S12B). Increasing the number of replicates has a dramatic effect on the detection rate of genes with smaller fold changes. Reducing the fold-change threshold reduces the TPR independently of replicate number for all the tools except *DEGSeq* (Fig. 2A–D). The reduced TPR associated with a reduced fold-change threshold can be recovered by increasing the replicate number. For example, achieving an ∼85% detection rate with *edgeR (exact)* for fold-change thresholds of *T* = 1, 0.3, and 0 requires ∼9, 11, and 26 replicates, respectively (Fig. 1B,C). For all the tools except *DEGSeq*, the TPR performance as a function of fold-change threshold has two distinct linear regions: a shallow linear regime at high-*T* and a steeper region at low-*T* (Fig. 1C; Supplemental Figs. S2, S3, S5–S12C). The transition between these two regions is a function of both the tool and the number of replicates. For *edgeR* (exact) with *n*_*r*_ = 3, this transition fold-change threshold is ∼0.5 and drops to ∼0.25 and ∼0.15 for *n*_*r*_ = 10 and 30, respectively (Fig. 1C). These transitions represent an optimal fold-change threshold to filter the data by, to maximize both the quality and the utility of the data.

The best performing tools, *DESeq, DESeq2, EBSeq, edgeR*, and *limma*, successfully control their FPR, maintaining it consistently close to or below 5% irrespective of fold-change threshold or number of replicates (Figs. 1B,C, 2; Supplemental Figs. S5, S7, S9B,C), highlighting again that the primary effect of increasing replicate number is to increase the sensitivity of these tools, converting false negatives to true positives (Fig. 1D; Supplemental Figs. S5, S7, S9D). Other tools are not so successful in this regard but a detailed interpretation of the FPR from this test is complicated by the fact that each tool is tested against its own gold standard. A more robust method for probing the FPR performance of DGE tools is presented below.

### Tool consistency with high replicate data

**FIGURE 3.**
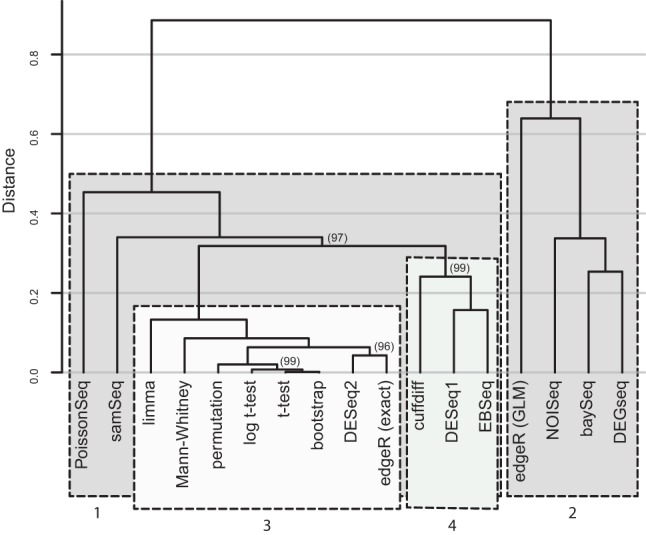
Hierarchical clustering of eleven RNA-seq DGE tools and five standard statistical tests using all of the full clean data set comprising 42 WT and 44 Δ*snf2* replicates. For each tool, or test, a 7126-element long vector of 1's and 0's was constructed representing whether each gene in the annotation was called as SDE (adjusted *P*-value or FDR threshold ≤0.05) by the tool or not. The vectors for each tool and test were then ordered by the gene id and hierarchically clustered by Correlation distance with complete linkage using the R package *pvclust*. Approximately unbiased *P*-value percentages (bracketed values) calculated for each branch in the clustering represent the support in the data for the observed sub-tree clustering. AU% > 95% are strongly supported by the data. AU% values are not shown for branch points where AU% = 100 for clarity. The outlier clustering of *baySeq*, *DEGSeq*, edgeR (GLM), and *NOISeq* suggest that these tools are clearly distinct from the other tools. Combined with the tool performance data shown in Figure 2, this suggests that, given a large number of replicates, the tools and tests in Cluster 1 are reliably and reproducibly converging on a similar answer, and are likely to be correctly capturing the SDE signal in the data.

The DGE tool performance tests described here assume that, given enough replicates, the tools converge on the true underlying differential expression signal in the data. This assumption was tested by clustering the DGE measurements for each tool's “gold standard” along with the results from five additional simple statistical tests applied to the same data (see Materials and Methods for a detailed description of the statistical tests). For each tool or test, a 7126-element long vector of 1s and 0s was constructed representing whether each gene in the annotation was called as SDE (adjusted *P*-value or FDR threshold ≤0.05) by the tool or not. The vectors for each tool or test were ordered by gene id and then hierarchically clustered by correlation distance with complete linkage (Fig. 3) using the R package *pvclust* (Fig. 3; Suzuki and Shimodaira 2006). *pvclust* uses bootstrapping to compute the statistical significance of subclusters within the dendrogram. Approximately unbiased *P*-value percentages (AU%—Fig. 3, bracketed values) calculated for each branch in the clustering are an indication of how robust each branch is to sampling error. Three widely used tools (*DESeq2*, *edgeR [exact]*, and *limma*, Table 1) are tightly grouped in a robust cluster with the standard statistical tests (Fig. 3, cluster 3). *cuffdiff*, *DESeq*, and *EBSeq* cluster tightly and are distinct from cluster 3 (Fig. 3, cluster 4). Despite the separation between these clusters being significant at the ∼3% level, this is the weakest clustering observed in the tree, suggesting that these tools and tests are converging on approximately the same answer, given a large number of replicates. Several of the standard statistical tests are nonparametric (Mann-Whitney, permutation and bootstrap) and use very different underlying methods compared to the tools in this cluster, indicating that the agreement of techniques within this group is not the result of a similar underlying methodology, but is likely reflective of the true differential expression signal in the data. *NOISeq*, *DEGSeq*, *baySeq*, and *edgeR (*generalized linear model; hereafter GLM) form a distinct independent cluster (Fig. 3, cluster 2) suggesting that these tools reach a considerably different result to those in Cluster 1.

### Testing tool false positive rates

**FIGURE 4.**
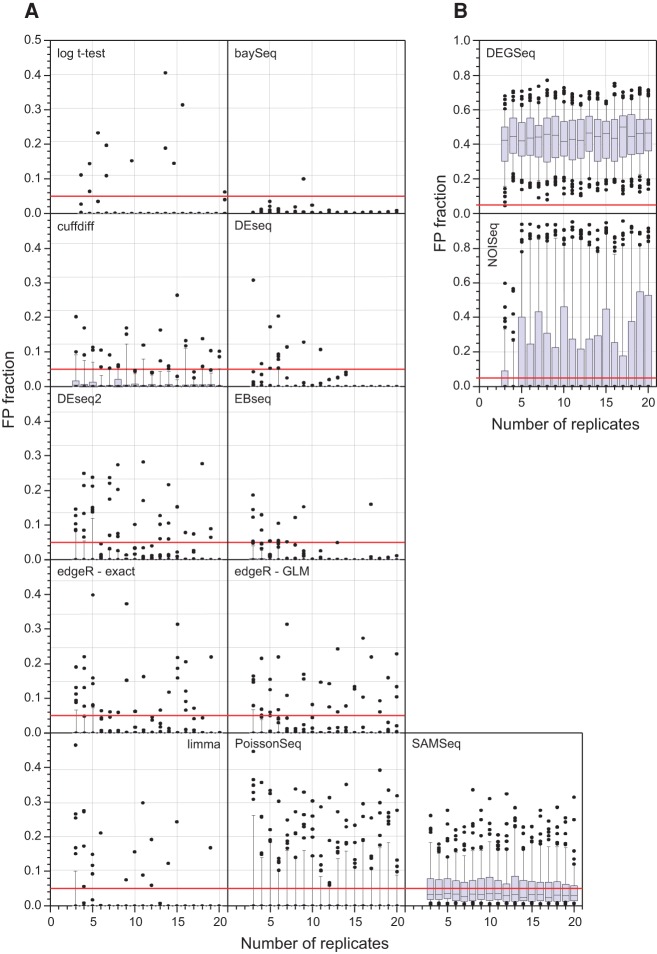
Testing false positive rate (FPR) performance: Each tool was used to call significantly differentially expressed (SDE) genes based on two artificial “conditions,” each constructed only from WT biological replicates. Genes identified as SDE are, by definition, false positives. The box plots show the median, quartiles, and 95% data limits on the FPR for 100 bootstrap iterations of each of the eleven tools and the log *t*-test for *n*_*r*_ = 3,4,..,20. The red line highlights a 5% FPR. (*A*) *y*-axis scale 0–0.5; (*B*) *y*-axis scale 0–1.0. In most cases the tools perform well for each bootstrap iteration, with only a small number of iterations showing a FPR > 5%. *DEGSeq*, *NOISeq*, and *SAMSeq* consistently show a higher and more variable FPR, suggesting that they are struggling to control their FPR adequately.

Perhaps the most important performance measure for RNA-seq differential expression tools is their false detection rate. The large number of replicates in this study permits a simple test of the FPR for each of the tools. Two sets of *n*_*r*_ replicates were randomly selected (without replacement) from the WT condition. Under the null hypothesis that there is no expression change between these two sets, every gene identified as SDE is, by definition, a false positive. For each bootstrap run, the fraction of the total gene set identified as SDE was computed. The distribution of this false positive fraction as a function of the number of replicates, for each differential expression tool, is shown in Figure 4. This approach shows that *DEGSeq, NOISeq*, and *SAMSeq* perform poorly even with a large number of replicates. *DEGSeq*, in particular, has poor false positive performance with every bootstrap iteration identifying >5% of all genes as false positives (FPs) and a median FPR of ∼50% irrespective of the number of replicates. Approximately 10% of *cuffdiff*, *PoissonSeq*, and ∼40% of *SAMSeq* bootstrap iterations identify >5% of all genes as FPs, suggesting that these tools are also not controlling their FPR well. *BaySeq*, *DESeq*, and *EBSeq* perform particularly well in this test with *edgeR*, *DESeq2*, and *limma* also performing adequately.

## DISCUSSION

In this work, the performance of eleven popular RNA-seq DGE tools has been evaluated using a highly replicated two-condition RNA-seq experiment designed specifically for the purpose of benchmarking RNA-seq DGE tools on genuine biological replicate data. Five of the 11 tools, *EBSeq*, *edgeR (exact)*, *DESeq*, *DESeq2*, and *limma* show excellent performance in the tests presented here. Reassuringly, *edgeR* and *DESeq* are the most widely used of the tools tested here as measured by citations (Table 1), suggesting that the majority of the RNA-seq DGE analyses in the literature are using the most appropriate tools for the job. An additional important feature of these five tools (run in GLM mode) is that they allow confounding experimental factors to be specified for DGE permitting them to be used even with challenging data sets. Where it is important to capture as many of the truly SDE genes as possible but with a low number of replicates (i.e., *n* ≲ 12), the data presented here suggest *edgeR (exact)* or *DESeq2* in preference to the other tools due to their superior TP identification rate and well-controlled FDR at lower fold changes. All the tools perform well for experiments with sufficient numbers of replicates to ensure that the majority of the true SDE is already being captured (i.e., *n* ≳ 12); however, the marginally better FPR performance of *DESeq* suggests it should be the tool of choice in this regime. Conversely, *baySeq*, *cuffdiff, DEGSeq, NOISeq, PoissonSeq*, and *SAMSeq* all show inferior performance in one or more areas. Table 2 summarizes the recommendations for choosing RNA-seq DGE tools, based on the results of these benchmarking tests.

**TABLE 2.**
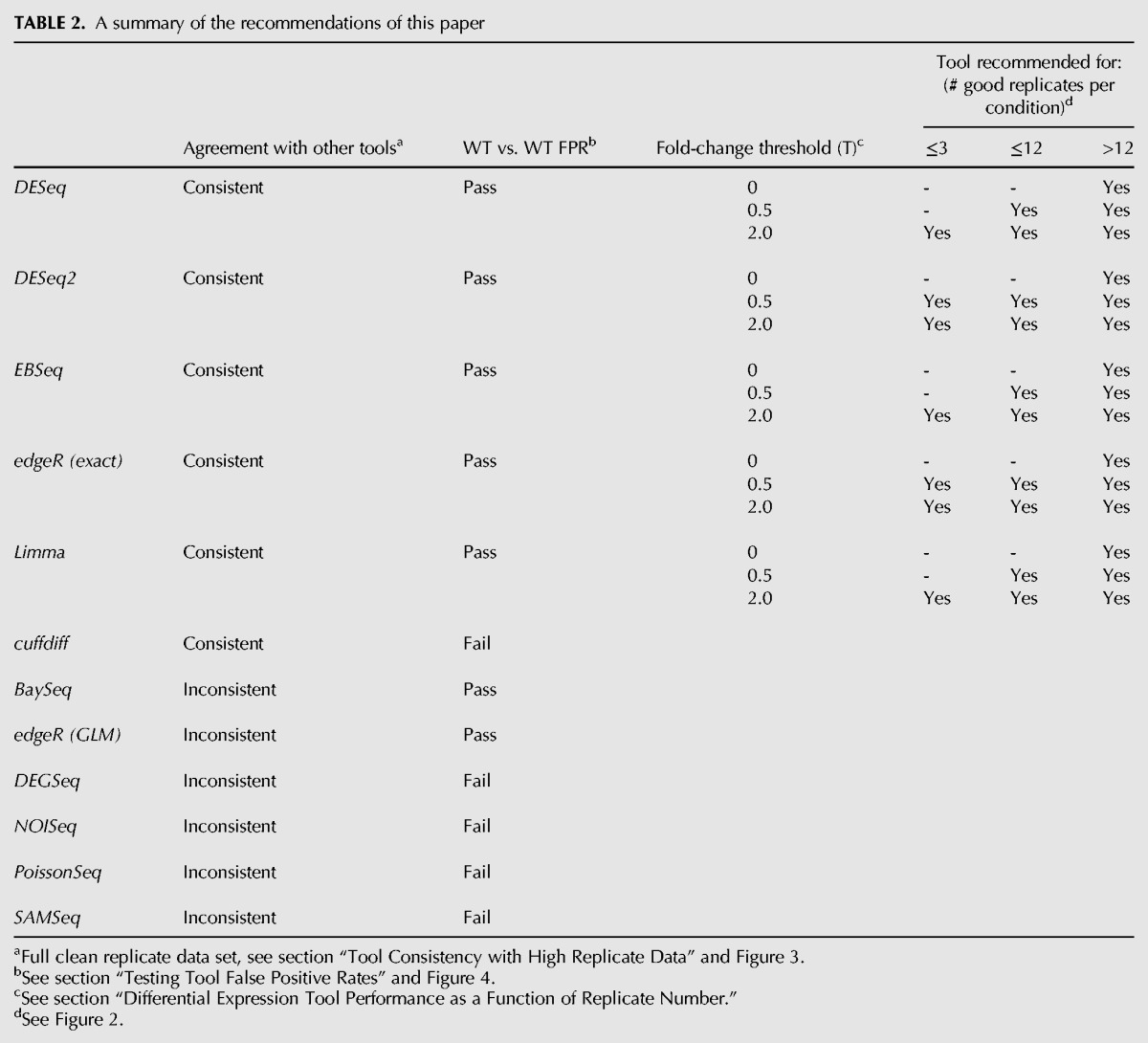
A summary of the recommendations of this paper

It is clear from the benchmarking runs that even the best tools have limited statistical power with few replicates in each condition, unless a stringent fold-change threshold is imposed (Fig. 2). For all the tools the FPR is approximately constant regardless of fold-change threshold, suggesting that controlling the FNR rather than the FPR is the primary justification for imposing this limitation. The variation intrinsic to any experimental procedures and protocols will result in a hard lower limit on the detectable fold changes for biologically relevant DGE. Unfortunately, it is not possible to calculate this limit here using the gene count data alone since it requires prior knowledge of actual fold changes to measure the impact of experimental variance. *DESeq2* includes an option to specify a fold-change threshold for the null hypothesis being tested. In this mode the tool tests whether the measured gene fold changes are consistent with being below this threshold value (rather than being consistent with zero), providing a natural mechanism for incorporating a fold-change threshold in a statistically meaningful way. As expected, this reduces the number of genes called as SDE. Setting *n*_*r*_ = 10 and running 100 *DESeq2* bootstraps, the number of SDE genes called is reduced from 3470 to 1277 by including a null hypothesis testing the fold-change threshold of 0.5.

When designing an RNA-seq experiment with the primary goal of identifying those SDE genes that change by more than a factor of two (*T* = 1), three clean replicates per condition may be sufficient. However, this is not the same as conducting the experiment with a total of three replicates, because there is a significant minority chance that one or more replicates within each condition should be rejected (see Gierliński et al. 2015). Conversely, for biological questions in which identifying the majority of the DE genes is important, a low-replicate experiment may not provide a sufficiently detailed view of the differential expression to inform the biology accurately. In these situations, it would be prudent to obtain at least 12 clean replicates per condition allowing the identification of ≳90% of the truly SDE genes with *T* ≳ 0.3 by any of the tools presented here. It is worth recalling that identifying a gene as SDE does not necessarily equate to identifying it as biologically significant and that it is important to consider both the magnitude of the measured fold change and existing biological knowledge alongside the statistical significance when inferring a biological significance for the results of DGE experiments.

The experiment performed here is likely to be a best-case scenario and thus represents the upper limit in performance of the tools tested. *S. cerevisiae* is one of the best-studied model organisms in biology, with a genome that is relatively small and well understood and few genes containing more than a single exon. Furthermore, the experiment contains no tissue-specific gene expression and the variation between biological replicates is small. In an experiment with samples from individuals, or samples with higher biological variation, the performance of all the DGE tools is likely to be worse. Similarly, for experiments using an organism with a more complex transcriptome, the performance of all the DGE tools is likely to be worse due to the presence of multiple transcript isoforms, anti-sense noncoding RNA transcription, and incomplete or poorly known annotations, particularly for 5′ and 3′ UTRs (Schurch et al. 2014). Although the majority of current DGE tools, including the 11 analyzed here, rely on an existing genome annotation, the recently published DGE tool *derfinder* (Frazee et al. 2014) examines differential expression for any region of a genome without annotations by analyzing differential expression at base pair resolution and grouping adjacent regions with similar signals. Such annotation-free differential expression tools may well represent the future for differential gene expression studies with RNA-seq data since they have the potential to mitigate the impact of genome annotation on detection of differential expression.

To the best of our knowledge, the experiment presented here is the most highly replicated RNA-seq data set to date and the only one specifically designed for testing the process of calling differential expression. As such, it will be a useful resource for the bioinformatics community as a test-bed for tool development, and for the wider biological science community as the most detailed description of transcription in wild-type and Δ*snf2* mutant *S. cerevisiae*.

### Recommendations for RNA-seq experiment design

The results of this study suggest the following should be considered when designing an RNA-seq experiment for DGE:

At least six replicates per condition for all experiments.
At least 12 replicates per condition for experiments where identifying the majority of all DE genes is important.
For experiments with <12 replicates per condition; use *edgeR (exact)* or *DESeq2*.
For experiments with >12 replicates per condition; use *DESeq*.
Apply a fold-change threshold appropriate to the number of replicates per condition between 0.1 ≤ *T* ≤ 0.5 (see Fig. 2 and the discussion of tool performance as a function of replication).

## MATERIALS AND METHODS

### The Δ*snf2* mutant

*Saccharomyces cerevisiae* is one of the best-studied organisms in molecular biology with a relatively small transcriptome and very limited alternative splicing and was chosen in order to give us the simplest RNA-seq data possible. *SNF2* is the catalytic subunit of ATP-dependent chromatin remodeling SWI/SNF complex in yeast. *SNF2* forms part of a transcriptional activator and mutation in *SNF2* brings about significant changes in transcription (e.g., Neigeborn and Carlson 1984; Stern et al. 1984; Peterson et al. 1991; Hirschhorn et al. 1992; Peterson and Herskowitz 1992; Holstege et al. 1998; Sudarsanam et al. 2000; Becker and Horz 2002; Gkikopoulos et al. 2011; Ryan and Owen-Hughes 2011, and references therein).

### *S. cerevisiae* growth conditions and RNA extraction

The *S. cerevisiae* strains used in the experiment were wild type (BY4741 strain, WT) and Δ*snf2* mutant in the same genetic background. Asynchronous WT and Δ*snf2* mutant strains were streaked out on rich media (YPAD) to get individual colonies. For 48 replicates in both strains, single colonies were inoculated to 15 mL cultures and cells were grown to an OD600 of 0.7–0.8 (corresponding to approximately 10^6^ cells) at 30°C. RNA was isolated using the hot-phenol method (Kohrer and Domdey 1991) and cleaned up using the RNeasy mini kit (Qiagen) protocol that uses Zymolyase for yeast cell lysis and DNase treatment to remove DNA contamination. The amount of total RNA extracted ranged from 30.3 to 126.9 µg per sample. Although the amount of RNA extracted was variable, the distributions were consistent with being drawn from the same population (Kolmogorov–Smirnov test, *P* = 0.16) indicating no bias in RNA content between WT and Δ*snf2* mutant samples.

### Library preparation, spike-in addition, and sequencing

The RNA-seq experiment described here implements a “balanced block design” in order to control for technical artifacts such as library batch effects (Kaisers et al. 2014), barcoding biases, and lane effects via randomization of the libraries (Colbourn and Dinitz 2007; Auer and Doerge 2010). Additionally, all the replicates include artificial RNA spike-in controls in order to allow external calibration of the RNA concentrations in each sample and of the measured fold changes between the two conditions (Jiang et al. 2011; Loven et al. 2012). The 96 samples were prepared in batches of 24 samples with 12 of each strain in each batch. Barcodes were preassigned randomly between the samples with barcode IDs 1–48 assigned to the Δ*snf2* mutant samples and 49–96 to the WT strain. For each batch the Illumina TruSeq protocol was used to prepare the sequencing library, with the addition of the ERCC spike-in standard (Ambion) (Jiang et al. 2011). Briefly, samples were poly(A) enriched with poly(dT) beads and 1 µL of 1:100 spike-in added to 19.5 µL of poly(A) enriched samples. Spike-in mix 1 was used with the Δ*snf2* mutant and mix 2 with WT. The RNA was then fragmented and subsequently underwent both first and second strand cDNA synthesis. The cDNA was then subjected to end repair, 3′ end adenylation, and barcode sequences were added. Finally, the un-barcoded adapters were ligated, templates purified and finally the samples were enriched via barcode-specific PCR primers. At this point the quality of the libraries was examined and passed before being diluted down to 10 nM and quantified (using fluorescence-based quantification) for accurate aliquoting for cluster generation and appropriate lane loading. Seven independent pools of the 96 barcoded samples were prepared and loaded onto seven lanes of an Illumina HiSeq 2000. Thus, each lane contains all 96 samples prepared in four batches with different spike-in mixes in each strain. The flow-cell was run for 51 cycles single-end.

### Read alignment and read-count-per-gene measurement

The lane data were demultiplexed and processed through Cassava pipeline v1.8 to generate 672 fastq files comprising seven technical replicates for each of the 96 biological replicates in the experiment. A total of ∼10^9^ reads were reported with each technical replicate having between 0.8 and 2.8 × 10^6^ reads. Aggregating the technical replicates across lanes results in ∼10^7^ reads per biological replicate. First pass quality control of the reads was performed with *fastQC* (http://www.bioinformatics.bbsrc.ac.uk/projects/fastqc) for each technical replicate. The reads from each technical replicate were then aligned to the Ensembl release 68 (Flicek et al. 2011) *S. cerevisiae* genome with *bowtie2* (*v2.0.0-beta7*) (Trapnell and Salzberg 2009) and *TopHat2* (*v2.0.5*) (Trapnell et al. 2009) using the following parameters: *--max-intron-length 1000 –min-intron-length 10 –microexon-search –b2-very-sensitive –max-multihits 1*. The aligned reads were then aggregated with *htseq-count* (v0.5.3p9, Anders et al. 2015) using the Ensembl v68 *S. cerevisiae* genome annotation to give total gene read counts for all 7126 gene features for each technical replicate. Finally, the read-count-per-gene measurements for each technical replicate were summed across sequencing lanes to give read-count-per-gene for each of the 96 biological replicates, and these were then used to identify poorly correlating “bad” replicates within the two conditions that were then subsequently removed from the analysis (see Gierliński et al. 2015 for a detailed description of this process). This resulted in a total of 42 WT and 44 Δ*snf2* biological replicates of “clean” read-count-per-gene data.

### Tool details and considerations for differential expression calculations

Most of the DGE tools assessed here calculate both a fold change (typically expressed as a logarithm to base 2 of the expression ratio, log_2_*FC*) and a statistical significance of differential expression for each gene. The fold change is based on the mean count across replicates in each condition, and for many of the tools this includes a calculation of sample-specific normalization factors based on the gene read-count data. For this study, the default normalization factors were used for each of the tools assessed. While there are differences between the normalizations used by these tools, it has been suggested that the details of which method is used to normalize the data does not significantly alter the downstream DGE results (Seyednasrollah et al. 2013). These normalization methods do, however, rely on the assumption that the majority of genes do not change their expression levels between conditions (e.g., Dillies et al. 2013). If this assumption is not satisfied, the measurements of both DGE fold change and significance are likely to be incorrect.

The statistical significances calculated by DGE tools are usually based on the null hypothesis of no expression change between the conditions. Calculating this significance typically relies on two key factors: (i) an assumption about the probability distribution that underlies the raw read-count measurements, and (ii) being able to accurately measure the mean count and variance for each gene. Different tools assume different forms for the underlying read-count distribution including the negative binomial (*baySeq*, *Cuffdiff*, *DESeq, DESeq2, EBSeq*, and *edgeR*), beta-binomial (*BBSeq*), binomial (*DEGSeq*), Poisson (*PoissonSeq*), and log-normal (*limma*) distributions. A few algorithms make no assumptions about the read-count distribution and instead take nonparametric approaches to testing for DGE (*NOISeq* and *SAMSeq*). Gierliński et al. (2015) show that for this data the majority of gene expression is consistent with both log-normal and negative binomial distributions except for the lowest expression genes, for which only the negative binomial distribution remains consistent with the data. For experiments with high numbers of replicates per condition (*n* ≳ 12), the mean and variance estimators can be accurately computed directly on the data. However, many RNA-seq DGE studies rely on a low number of replicates per condition (*n* ≲ 3), so several of the DGE tools (e.g., *DESeq*, *DESeq2, edgeR*, *limma*) compensate for the lack of replication by modeling the mean-variance relation and borrowing information across genes to shrink the given gene's variance toward the common model (Cui et al. 2005; De Hertogh et al. 2010; Robinson et al. 2010). The stabilized variance helps avoid some of the spurious false positives and negatives, but is strongly dependent on an assumed read count distribution and on the assumptions intrinsic to the normalization of the count data, namely that the large majority of the gene counts are not truly differentially expressed. For a full description of the measured individual gene read count distributions in these data, a comparison of these with the assumptions made by DGE tools, and the impact this has on the DGE results, see Gierliński et al. (2015). Given these methods' dependence on accurate mean and variance measurements, it is somewhat surprising that scientists would contemplate doing DGE analysis without replicated data, but for completeness we note that several DGE analysis tools advertise that they can work with a single replicate per condition (Anders and Huber 2010; Robinson et al. 2010; Tarazona et al. 2011).

### Bootstrap differential expression calculations

A utility pipeline was written to automate the process of running each DGE algorithm iteratively on *i* repeated subselections of clean replicates. Each subselection is comprised of *n*_*r*_ replicates chosen at random without replacement (that is, an individual replicate can appear only once within each subselection). This bootstrapping procedure includes applying the default normalization for each tool where relevant and possible (see section “Tool Details and Considerations for Differential Expression Calculations”) and the full output for each tool was stored in a local *sqlite* database, including the log_2_ transformed fold change and the statistical significance for every expressed gene in the *S. cerevisiae* annotation. Most of the tools return Benjamini–Hochberg (hereafter BH; Benjamini and Hochberg 1995) corrected *P*-values or FDRs as their measure of statistical significance. Genes with an adjusted *P*-value or FDR ≤ 0.05 were marked as “significantly differentially expressed” (SDE). Supplemental Figure S1 shows an example of the output mean log_2_*FC* and median *P*-value data for the tool *edgeR* (exact) with *n*_*r*_ = 3.

From these data, TPRs, TNRs, FPRs, and FNRs for each tool were computed as a function of the number of replicates, *n*_*r*_, for four arbitrary absolute log_2_ fold-change thresholds, T∈{0,0.3,1,2}. A reference fold change was used for deciding whether each gene falls above the threshold *T* because the measured values of mean $\left|\log_{2}FC\right|$ calculated for a gene varies considerably with both the tool being used and *n*_*r*_. These reference fold changes were defined independently of the tools by applying *DESeq* normalization (Anders and Huber 2010) to the read-count-per-gene data from the full clean set of biological replicates for each condition and then taking the log_2_ transformed ratio of the mean normalized read-count-per-gene for each condition. For each individual DGE calculation within a bootstrap run (i.e., an individual differential expression calculation with a specific tool with a given *n*_*r*_), each gene was called as true/false positive/negative by comparing whether it was called as SDE in the bootstrap run, and whether it was called as SDE in the corresponding tool-specific “gold standard.” Then, taking each fold-change threshold in turn, the mean of the number of true/false positives/negatives (TP, TN, FP, FN) for genes with reference fold changes above this threshold was calculated across all the individual DGE calculations within a bootstrap run. This results in a TPR, TNR, FPR, and FNR for a tool, for a given *n*_*r*_ and for a given *T* (Equations 1–4):

(1) $$\mathrm{TPR}(n_{r},T)=\frac{\mathrm{TP}(n_{r},T)}{\mathrm{TP}(n_{r},T)+\mathrm{FN}(n_{r},T)}$$

(2) $$\mathrm{FPR}(n_{r},T)=\frac{\mathrm{FP}(n_{r},T)}{\mathrm{FP}(n_{r},T)+\mathrm{TN}(n_{r},T)}$$

(3) $$\mathrm{TNR}(n_{r},T)=\frac{\mathrm{TN}(n_{r},T)}{\mathrm{TN}(n_{r},T)+\mathrm{FP}(n_{r},T)}$$

(4) $$\mathrm{FNR}(n_{r},T)=\frac{\mathrm{FN}(n_{r},T)}{\mathrm{FN}(n_{r},T)+\mathrm{TP}(n_{r},T)}$$

Uncertainties in the resulting values were calculated by propagating the standard deviations of the numbers of TPs, TNs, FPs, and FNs across the calculations within each bootstrap run, to reflect the spread of calculated values due to the random sampling of replicates.

### Standard statistical tests for differential expression

When assessing the performance of each DGE tool on the full set of clean data, we compare the tools not only within themselves, but also to the following set of standard statistical tests. For the following mathematical descriptions, $x_{gk}=(x_{g1k},\;x_{g2k},\;\ldots ,\;x_{gn_{k}k})$ is a vector of *n*_*k*_ (clean) replicates for gene *g* and condition *k*, ${\bar{x}}_{gk}$ and $s_{gk}^{2}$are the mean and variance of this vector.

#### t-test

The null hypothesis in the *t*-test is that the given gene under two conditions has the same mean count, *H*_0_ : µ_*g*1_ = µ_*g*2_. We used the test statistic

(5) $$t_{g}=\frac{{\bar{x}}_{g1}-{\bar{x}}_{g2}}{\sqrt{s_{g12}^{2}\left(\frac{1}{n_{1}}+\frac{1}{n_{2}}\right)}}$$

with common variance estimator $s_{g12}^{2}=[(n_{1}-1)s_{g1}^{2}+(n_{2}-1)s_{g2}^{2}]/\nu$, and the number of degrees of freedom is *v* = *n*_1_ + *n*_2_ − 2.

#### Log-ratio t-test

This modified *t*-test is more appropriate for log-normally distributed data. The null hypothesis is lnµ_*g*1_ = lnµ_*g*2_. The test statistic,

(6) $$t_{g}=\frac{\ln{\bar{x}}_{g1}-\ln{\bar{x}}_{g2}}{\sqrt{\frac{s_{g1}^{2}}{n_{1}{\bar{x}}_{g1}^{2}}+\frac{s_{g2}^{2}}{n_{2}{\bar{x}}_{g2}^{2}}}},$$

is approximately distributed with *t*-distribution with *n*_1_ + *n*_2_ − 2 degrees of freedom (see Olsson 2005).

#### Mann-Whitney test

The Mann-Whitney (Mann and Whitney 1947—hereafter MW) test is a nonparametric test assessing if count rate in a gene under one condition tends to be larger than under the other. The null hypothesis is $H_{0}:\mathrm{Pr}(x_{gi1}>x_{gj2})=1/2$, for each pair of replicates *i* and *j*. *P*-values were calculated using normal approximation (Bellera et al. 2010) and taking ties into account (Sheskin 2004). The MW test relies on ranks, not actual data values, which makes it distribution-free. On the other hand, when every replicate in one condition is larger than every replicate in the other condition, the MW test will return the same *P*-value, regardless of how much the two conditions differ.

#### Permutation test

In the permutation test, counts from both conditions are pooled together (for each gene), ***x***_*g*_ = (***x***_*g*1_,***x***_*g*2_) and then randomly resampled *B* times without replacement from ***x***_*g*_, using the original sizes, *n*_1_ and *n*_2_. For the *b*-th random permutation $x_{g1}^{*}(b)$ and $x_{g2}^{*}(b)$ we find the test statistic, $D_{g}^{*}(b)={\bar{x}}_{g1}^{*}(b)-{\bar{x}}_{g2}^{*}(b)$, which is the difference between the means of the two sampled vectors. This is compared with the observed statistic $D_{g}={\bar{x}}_{g1}-{\bar{x}}_{g2}$. The test *P*-value is the fraction of cases where the resampled statistic exceeds the observed one, $\;p_{g}=\#\{|D_{g}^{*}(b)|>|D_{g}|\}/B$ (for more details, see Efron and Tibshirani 1993a). The advantage of the permutation test is that it does not make any assumptions about the underlying distribution, but rather models it directly from data. The disadvantage is that it requires many replicates to build this underlying distribution, as it is not applicable for a typical experiment with, say, three replicates.

#### Bootstrap test

The Studentized bootstrap test described by Efron and Tibshirani (1993b) was used here. It estimates probability distribution of the two populations with sample sizes *n*_*1*_ and *n*_*2*_, under the null hypothesis of the common mean. Data are resampled with replacement to estimate the significance level. For the *b*^*th*^ bootstrap, $x_{g1}^{*}(b)$ and $x_{g2}^{*}(b)$, the test statistic is

(7) $$t_{g}^{*}(b)=\frac{{\bar{x}}_{g1}^{*}(b)-{\bar{x}}_{g2}^{*}(b)}{\sqrt{s_{g12}^{*2}(b)\left(\frac{1}{n_{1}}+\frac{1}{n_{2}}\right)}},\;b=1,\;2,\;...\;B,$$

where the common variance estimator is $s_{g12}^{*2}(b)=[\left(n_{1}-1)s_{g1}^{*2}(b)+(n_{2}-1)s_{g2}^{*2}(b\right)]/(n_{1}+n_{2}-2)$. This is compared with the observed statistic (Equation 5). As in the permutation test, the test *P*-value is the fraction of cases where the resampled statistic exceeds the observed one, $\;p_{g}=\#\{|t_{g}^{*}(b)|>|t_{g}|\}/B$.

## DATA DEPOSITION

The data sets supporting the results of this article are available in the European Nucleotide Archive repository (ENA) (PRJEB5348, http://www.ebi.ac.uk/ena/data/view/ERX425102). All the code for this work is publicly available (https://github.com/bartongroup/profDGE48).

## SUPPLEMENTAL MATERIAL

Supplemental material is available for this article.

## Supplementary Material

Supplemental Material
