# Supplementary material for: How many biological replicates are needed in an RNA-seq experiment and which differential expression tool should you use?
Source: RNA. 2016 Jun;22(6):839–51. doi: 10.1261/rna.053959.115 (PMC4878611; doi:10.1261/rna.053959.115)
Supplement: Supplemental Material [file supp_22_6_839__index.html]

How many biological replicates are needed in an RNA-seq experiment and which differential expression tool should you use? — Supplemental Material 

# How many biological replicates are needed in an RNA-seq experiment and which differential expression tool should you use?

## Supplemental Material

**Files in this Data Supplement:**

- Supp\_FigureS7.pdf - pdf file
- Supp\_FigureS10.pdf - pdf file
- Supp\_FigureS2.pdf - pdf file
- Supp Figure S4.pdf
- Supp Figure S6.pdf
- Supp\_FigureS11.pdf - pdf file
- Supp\_FigureS5.pdf - pdf file
- Supp\_FigureS9.pdf - pdf file
- Supp\_FigureS3.pdf - pdf file
- Supp\_FigureS1.pdf - pdf file
- Supp\_FigureS8.pdf - pdf file
- Supp\_FigureS12.pdf - pdf file
- Supp\_Figure\_Legends.docx - docx file
