# Supplementary figures and images for: How many biological replicates are needed in an RNA-seq experiment and which differential expression tool should you use?
Source: RNA. 2016 Jun;22(6):839–51. doi: 10.1261/rna.053959.115 (PMC4878611; doi:10.1261/rna.053959.115)

# Supp. Fig. 7

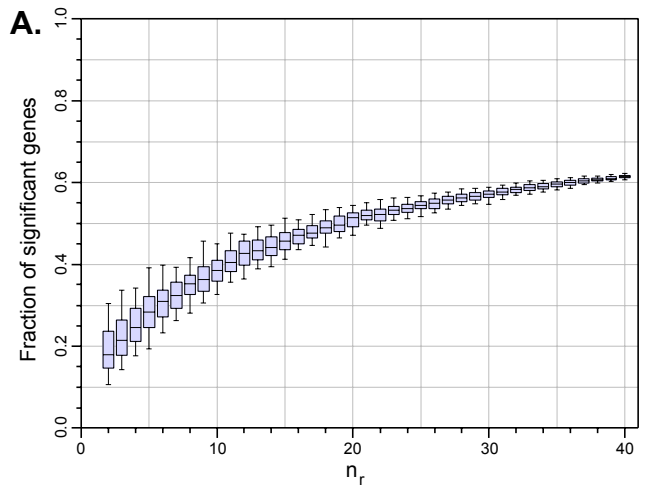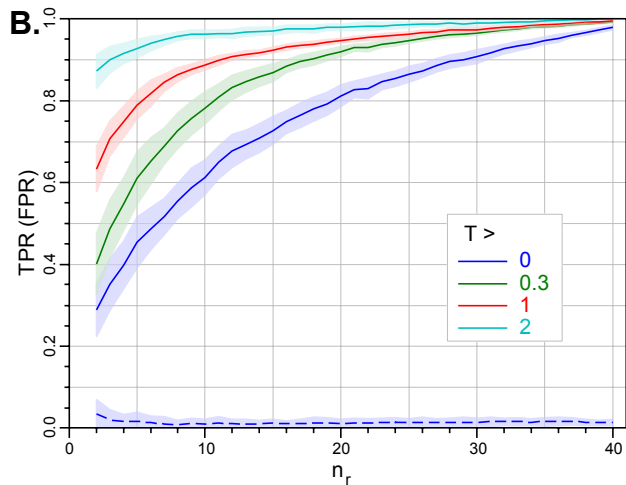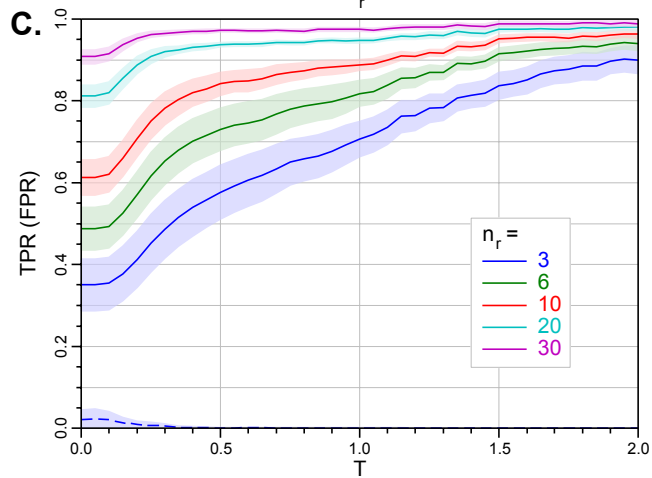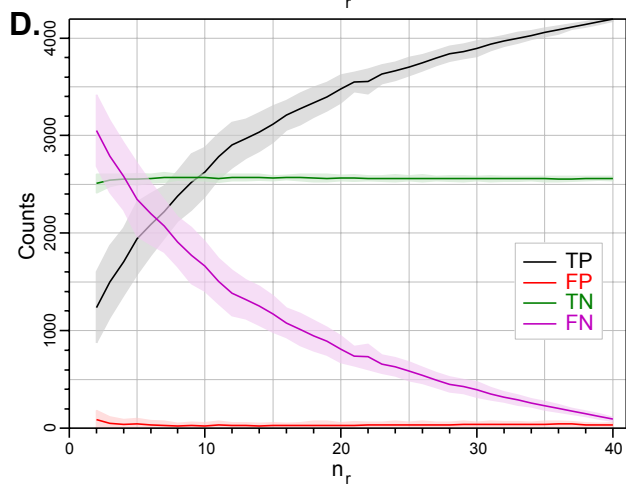

Supplement: Supplemental Material [file supp_053959.115_Supp_FigureS7.pdf]

# Supp. Fig. 10

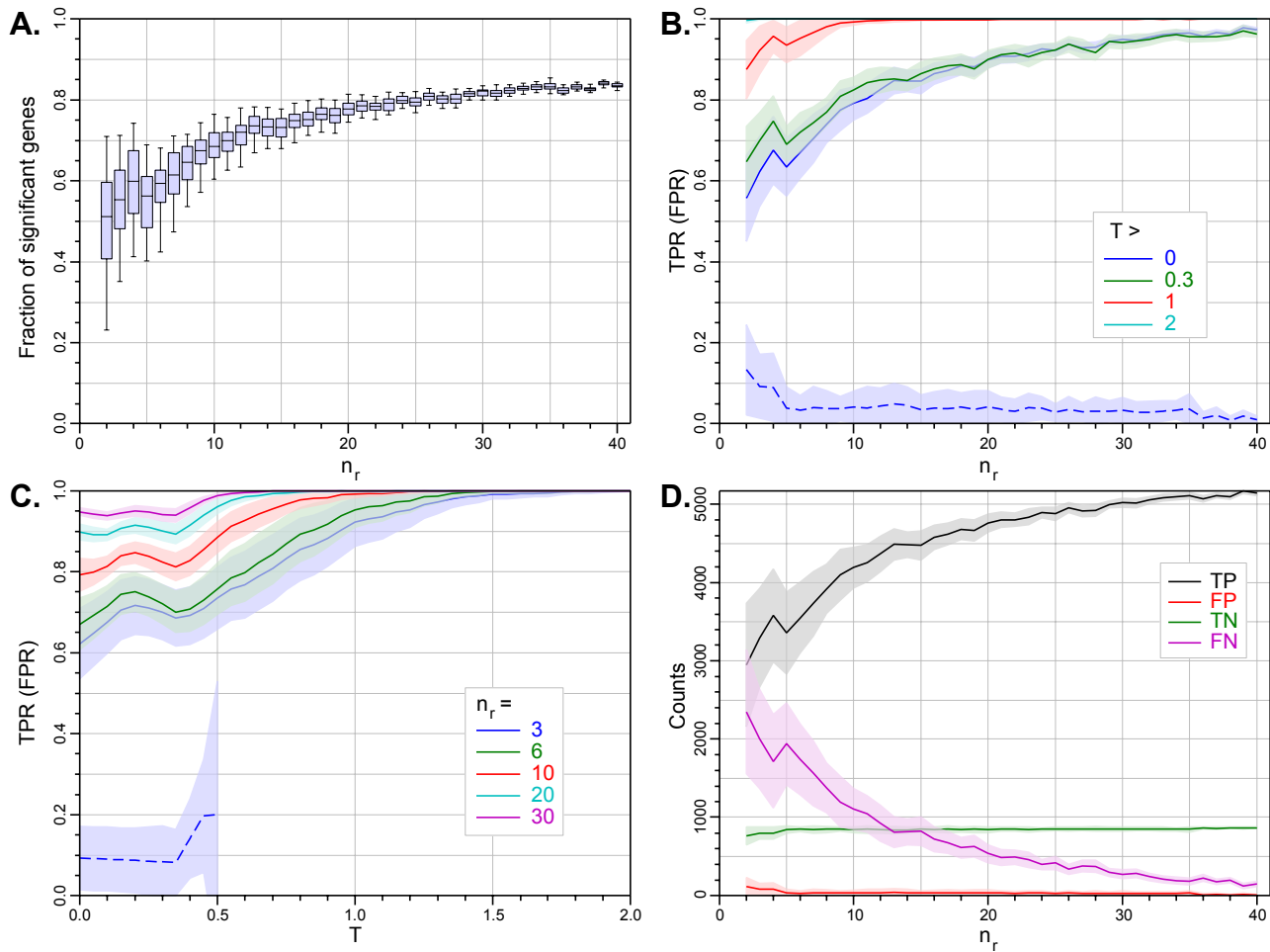

Supplement: Supplemental Material [file supp_053959.115_Supp_FigureS10.pdf]

## Supp. Fig. 2

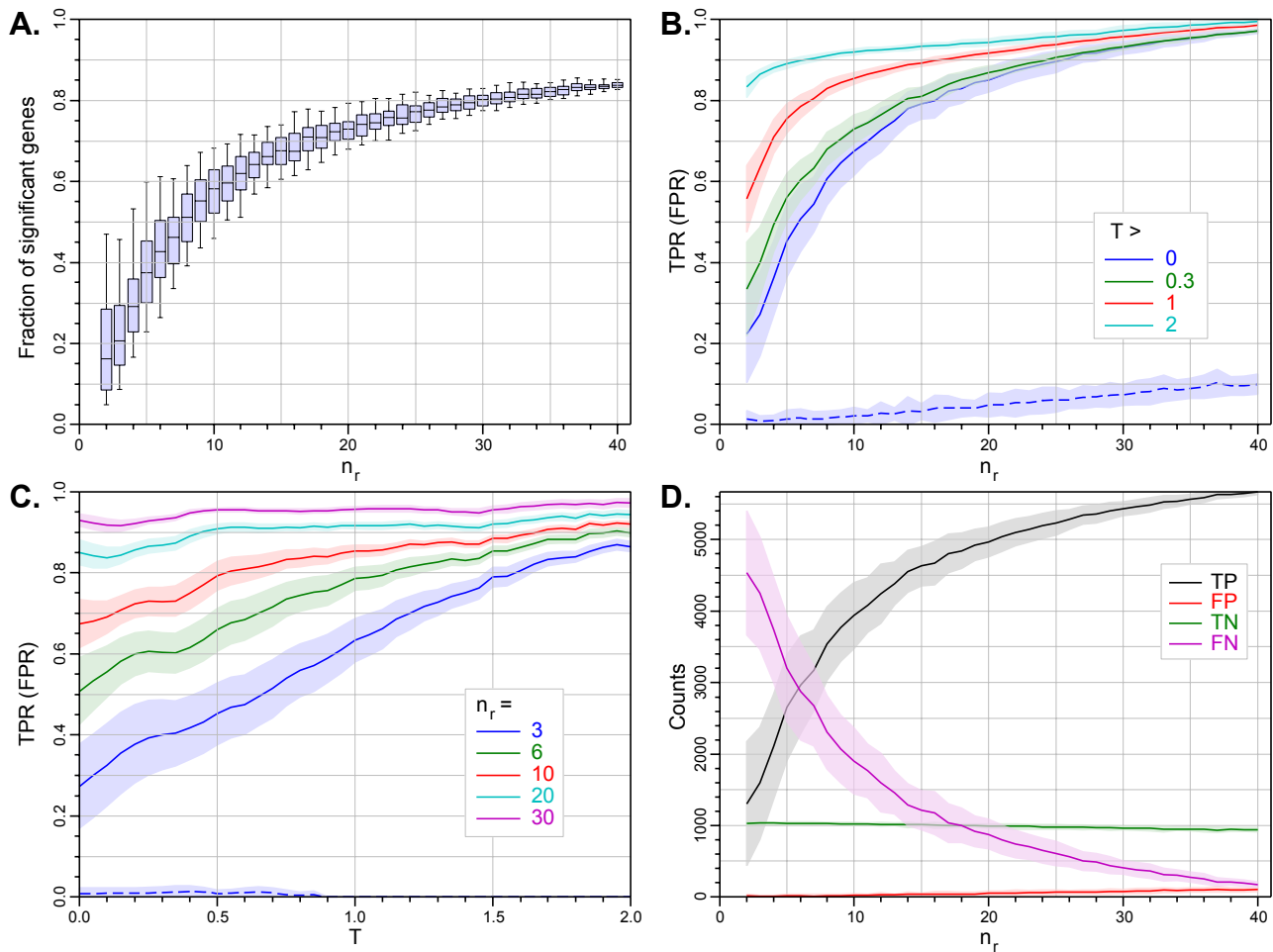

Supplement: Supplemental Material [file supp_053959.115_Supp_FigureS2.pdf]

## Supp. Fig. 4

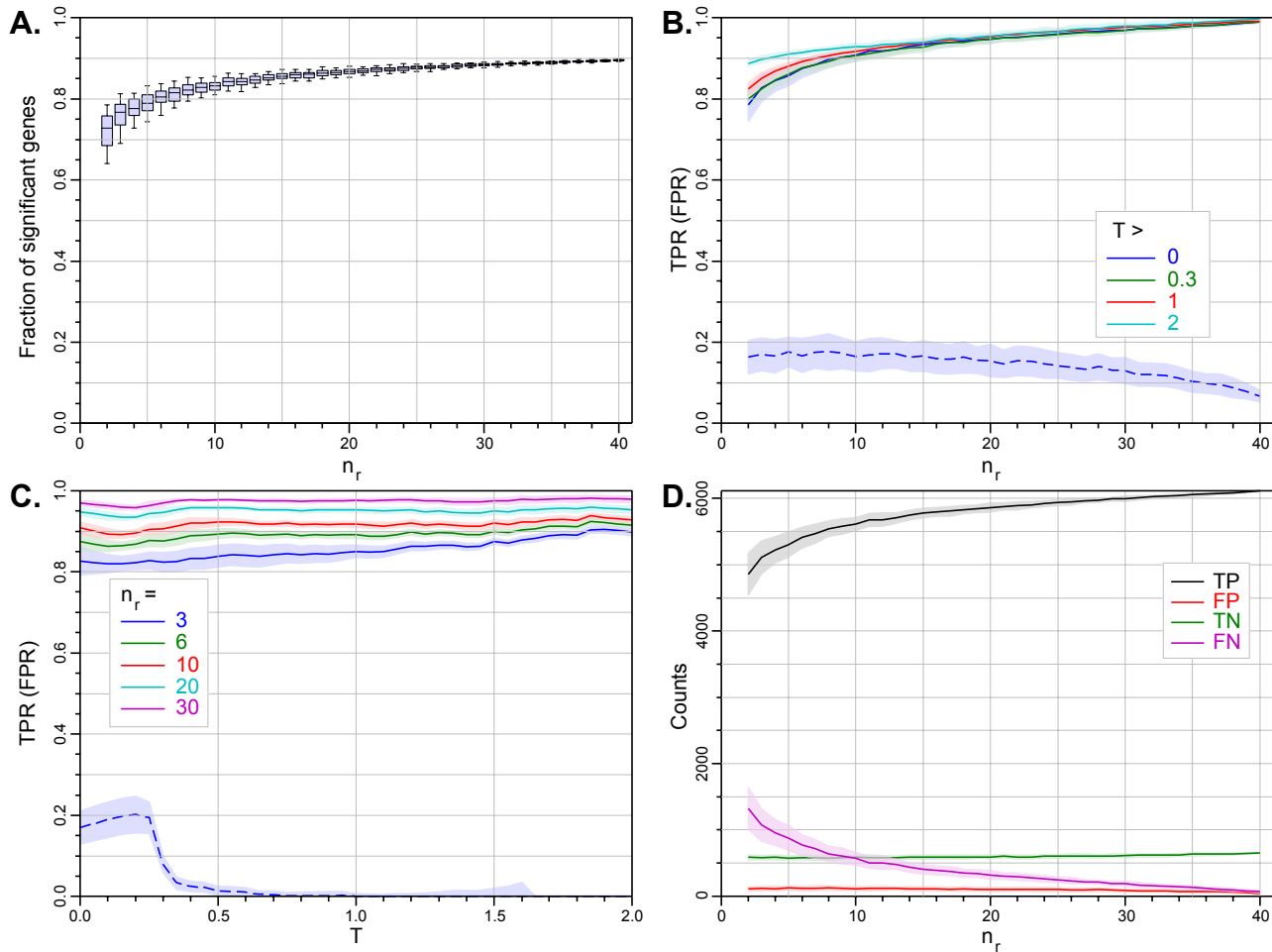

Supplement: Supplemental Material [file supp_053959.115_SuppFigureS4.pdf]

**Supp. Fig. 6**

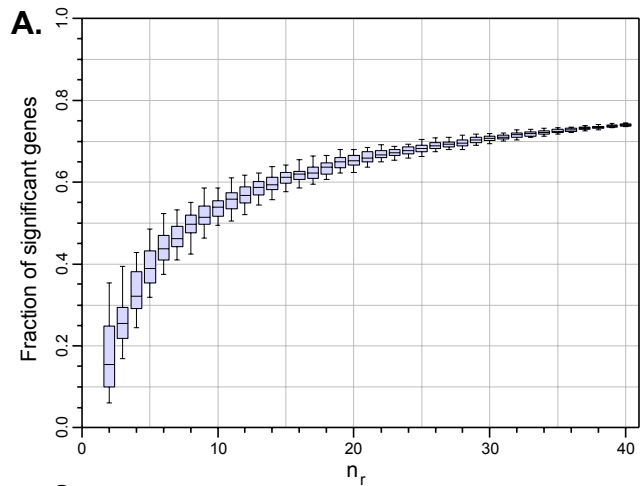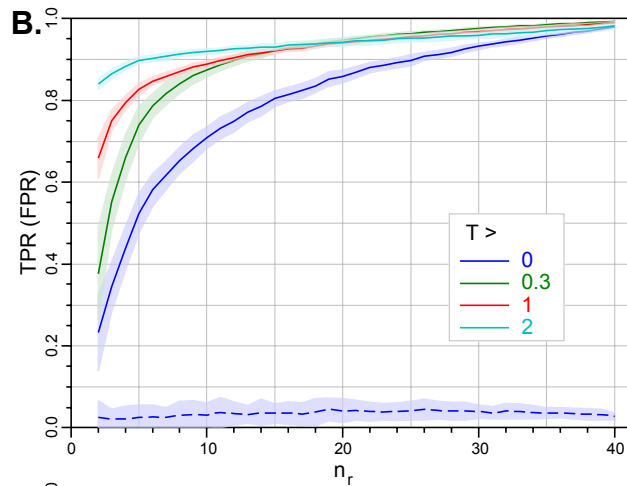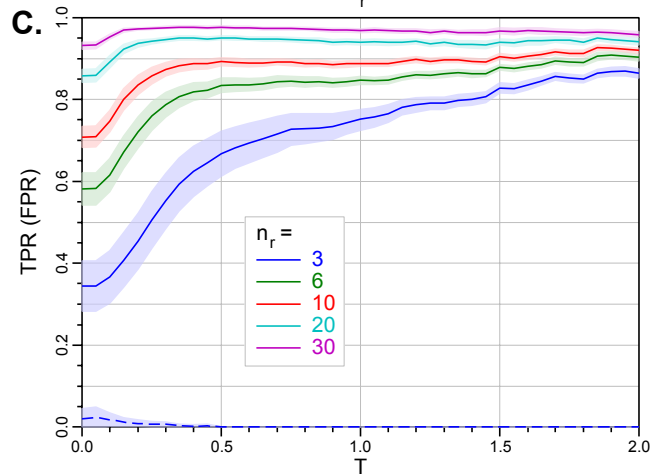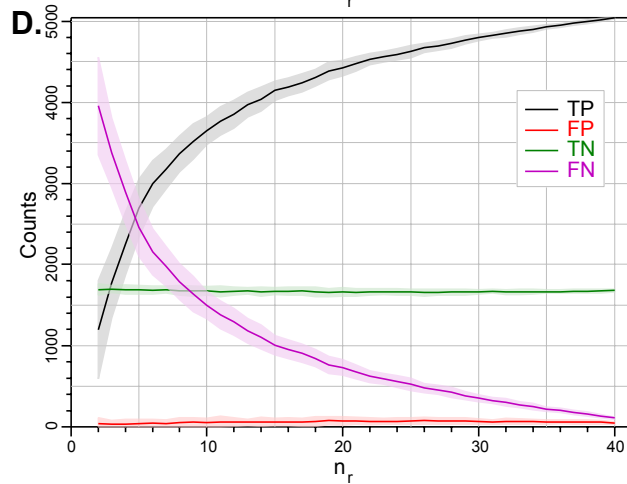

Supplement: Supplemental Material [file supp_053959.115_SuppFigureS6.pdf]

**Supp. Fig. 11**

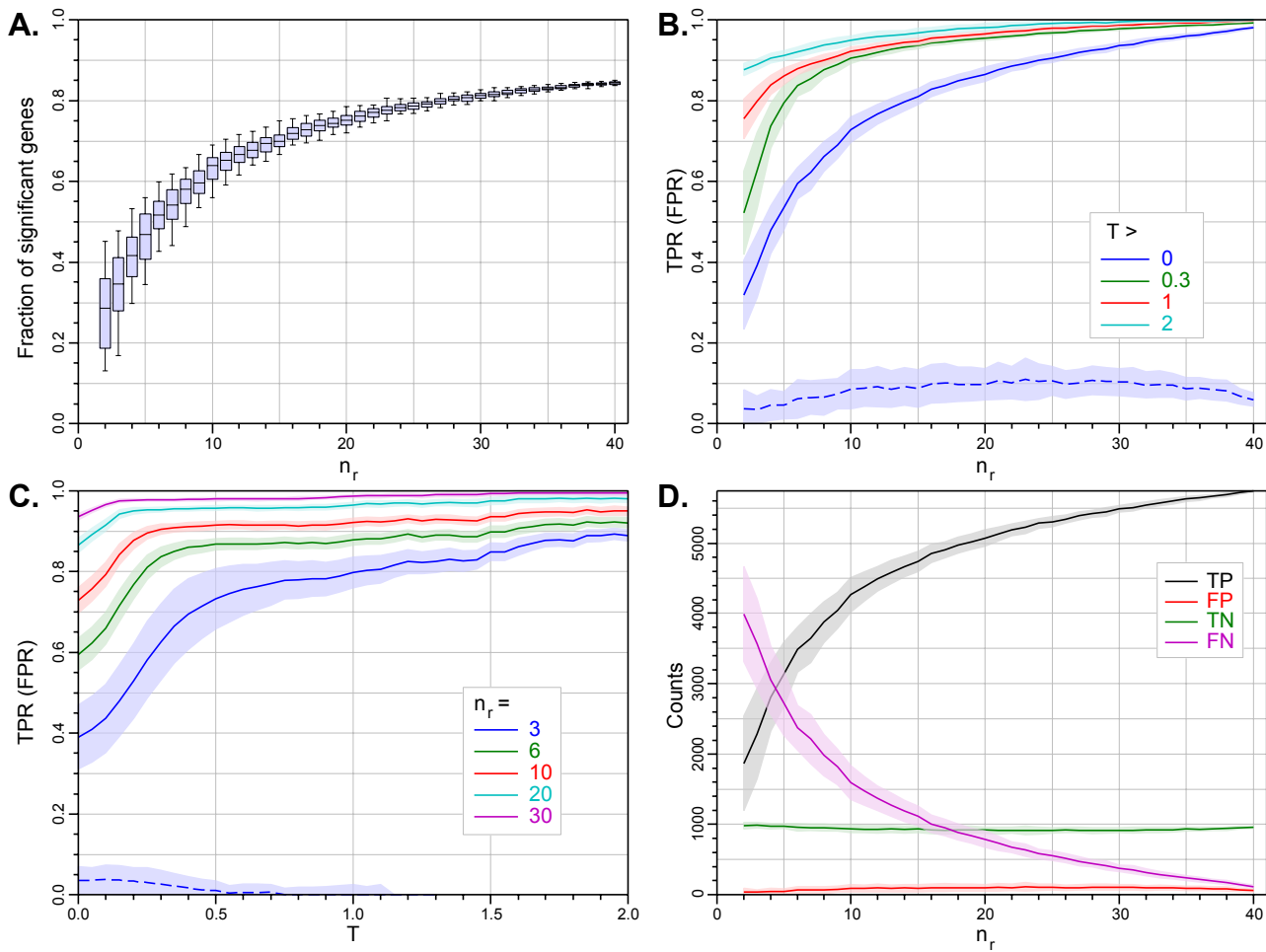

Supplement: Supplemental Material [file supp_053959.115_Supp_FigureS11.pdf]

# Supp. Fig. 5

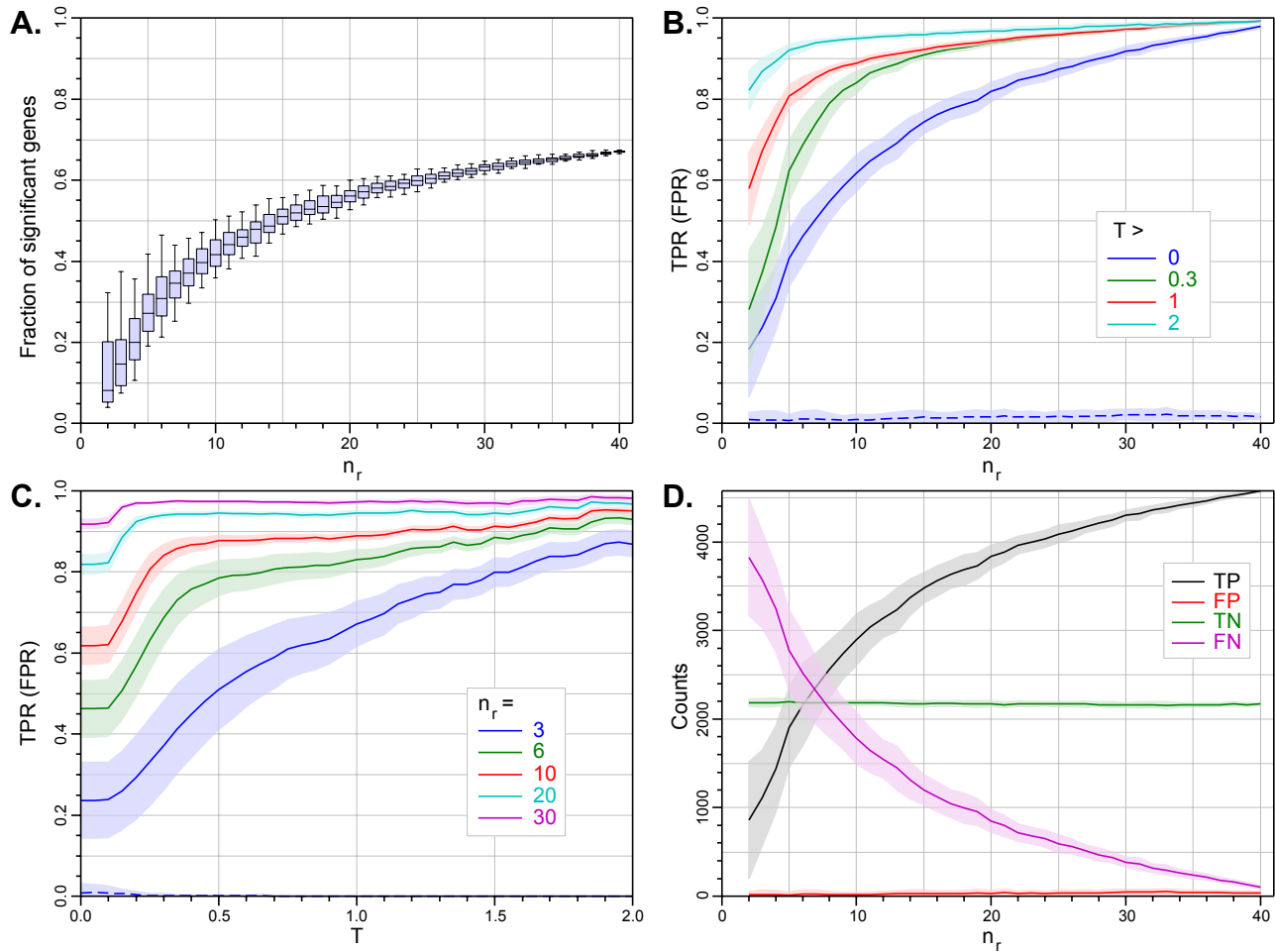

Supplement: Supplemental Material [file supp_053959.115_Supp_FigureS5.pdf]

**Supp. Fig. 9**

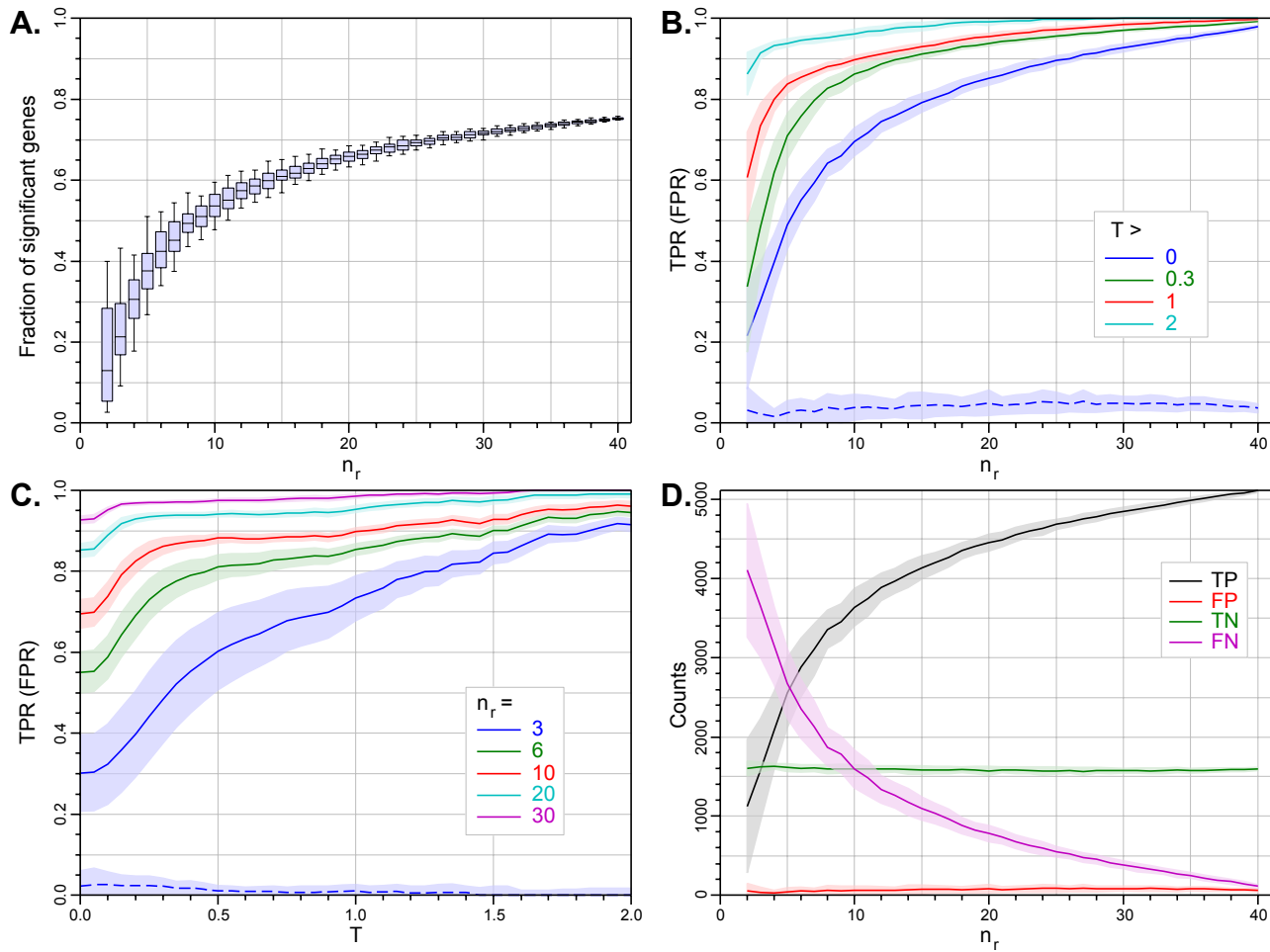

Supplement: Supplemental Material [file supp_053959.115_Supp_FigureS9.pdf]

## Supp. Fig. 3

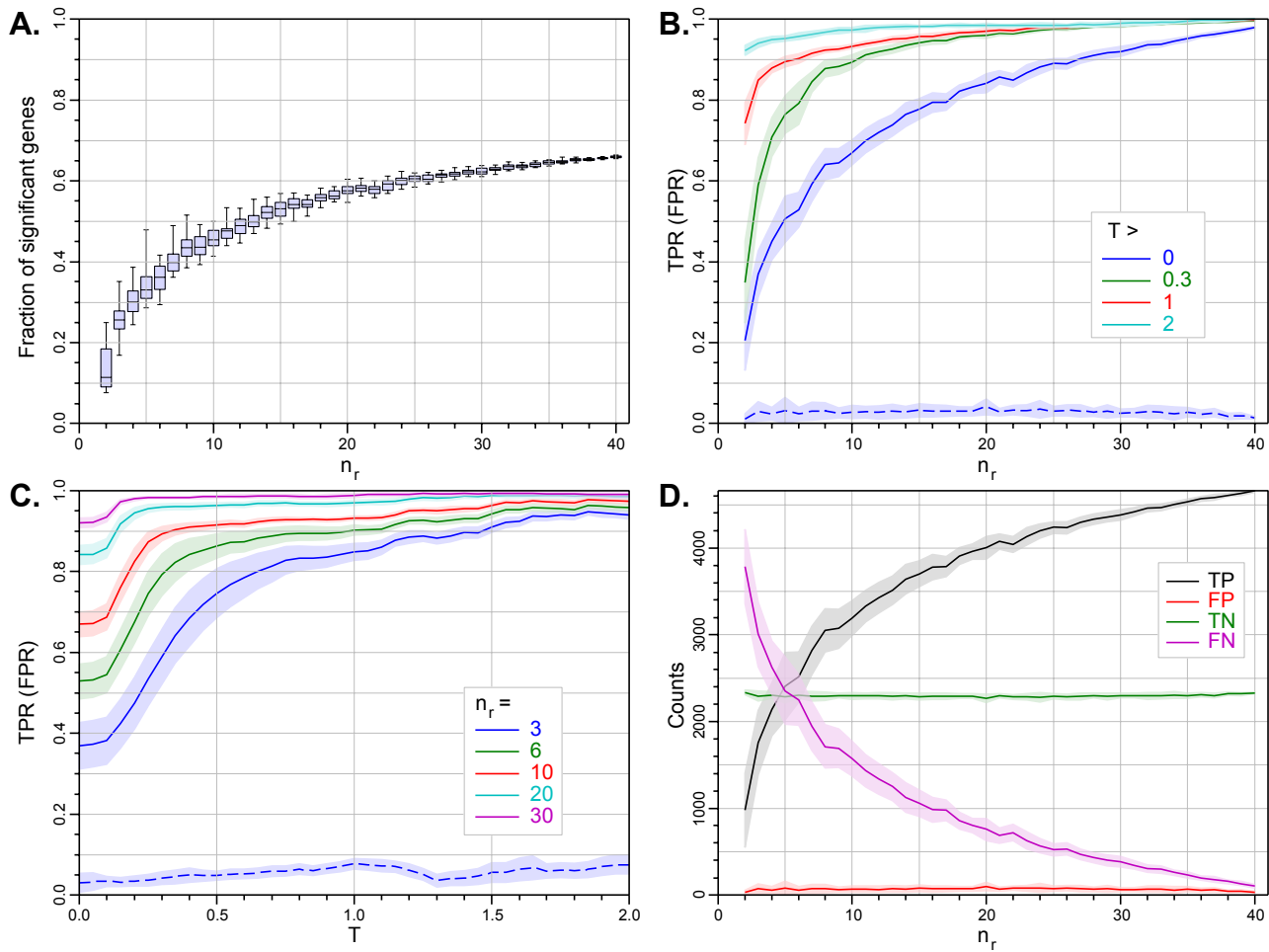

Supplement: Supplemental Material [file supp_053959.115_Supp_FigureS3.pdf]

Supp. Fig. 1

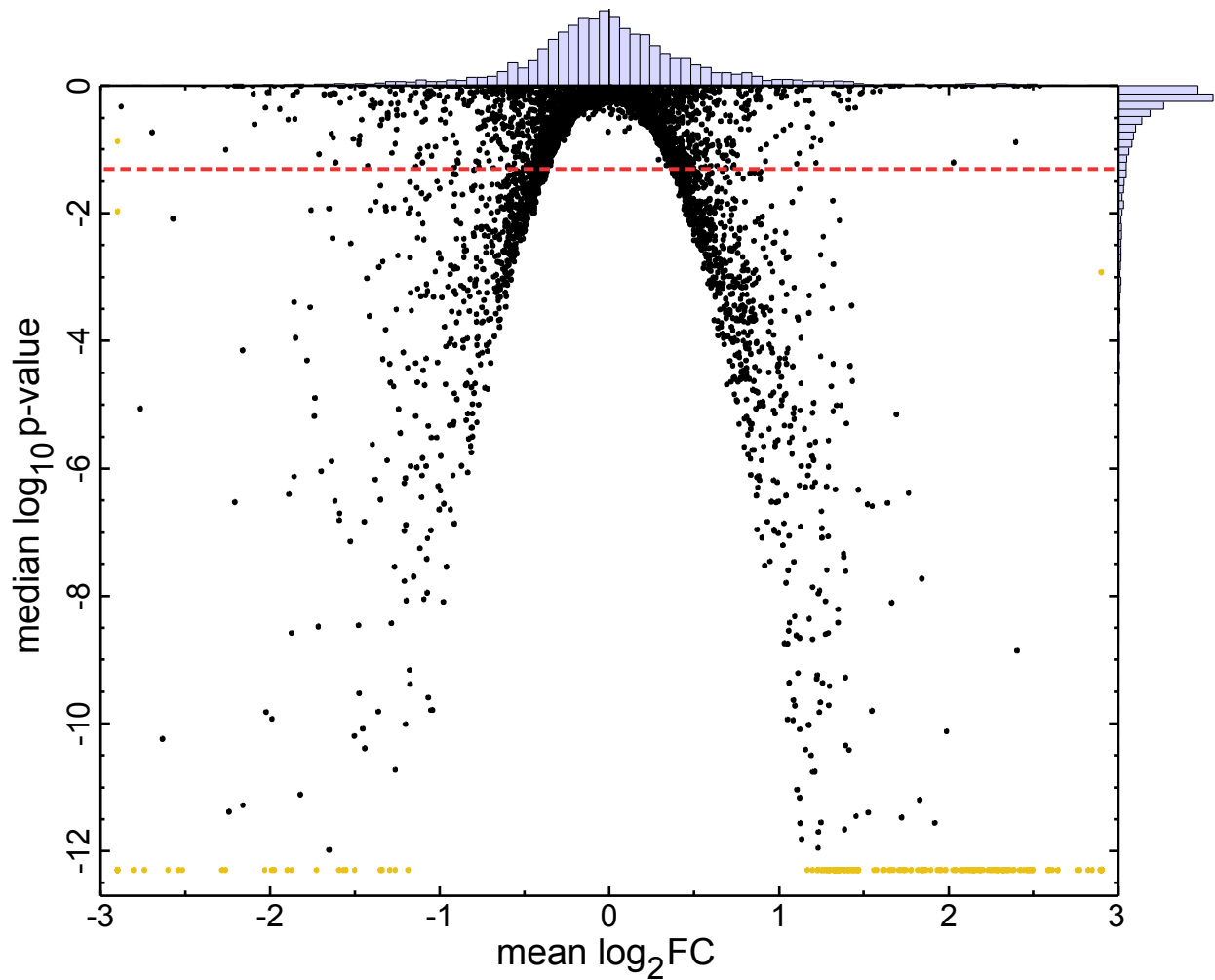

Supplement: Supplemental Material [file supp_053959.115_Supp_FigureS1.pdf]

Supp. Fig. 8

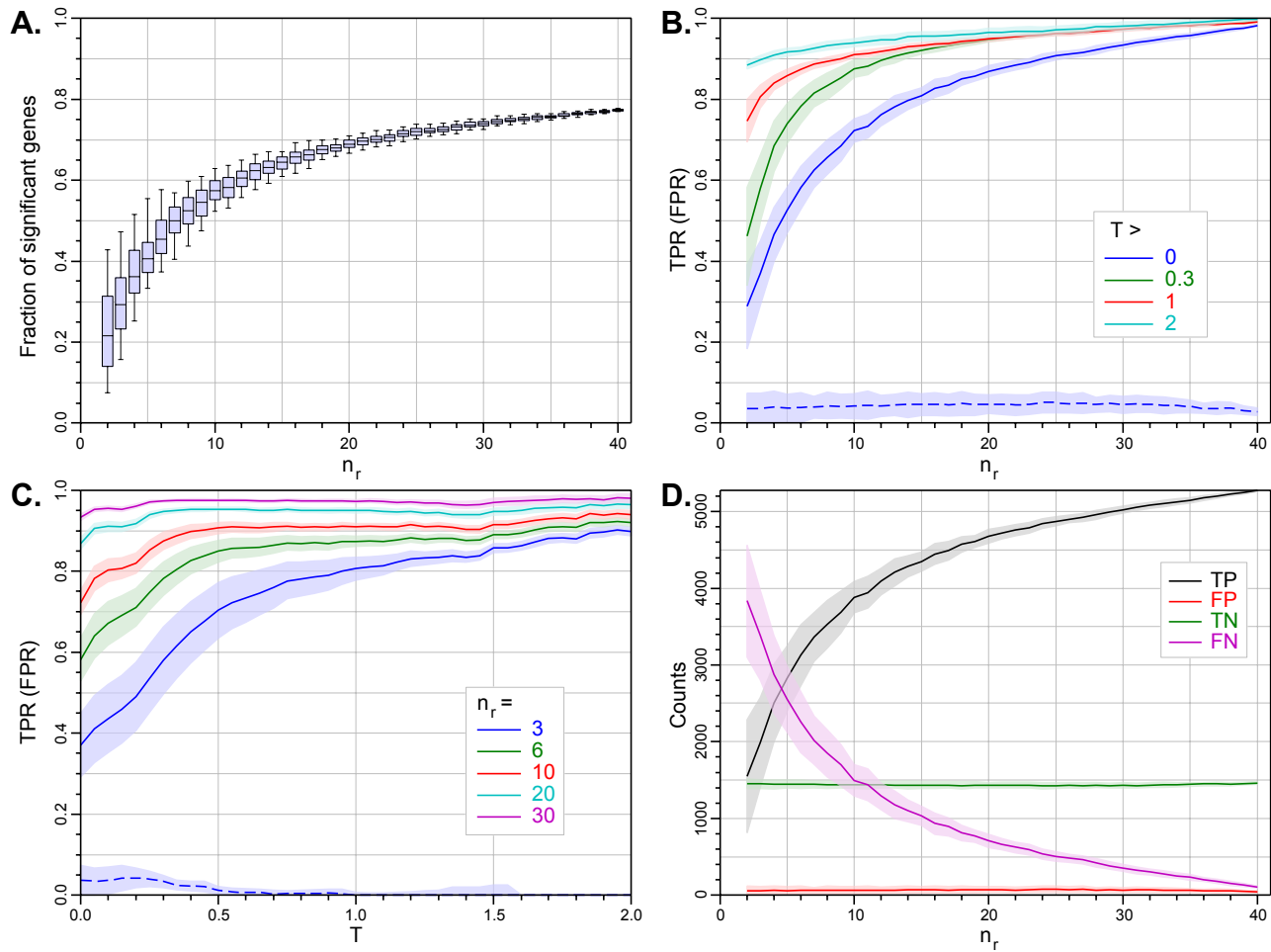

Supplement: Supplemental Material [file supp_053959.115_Supp_FigureS8.pdf]

## Supp. Fig. 12

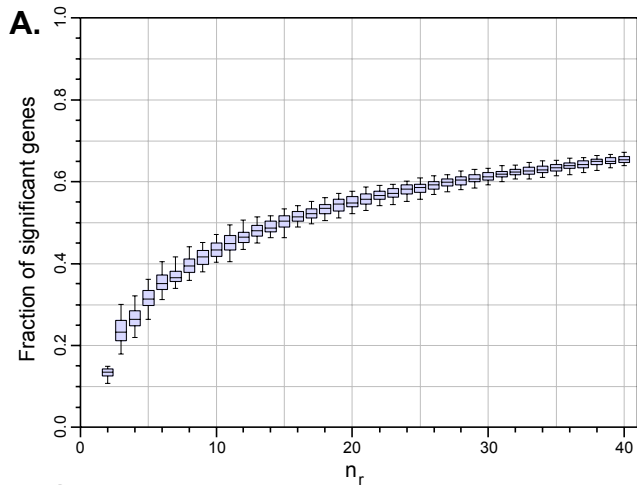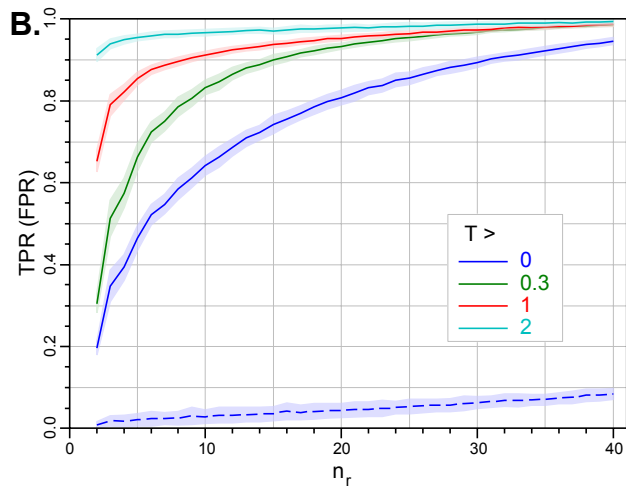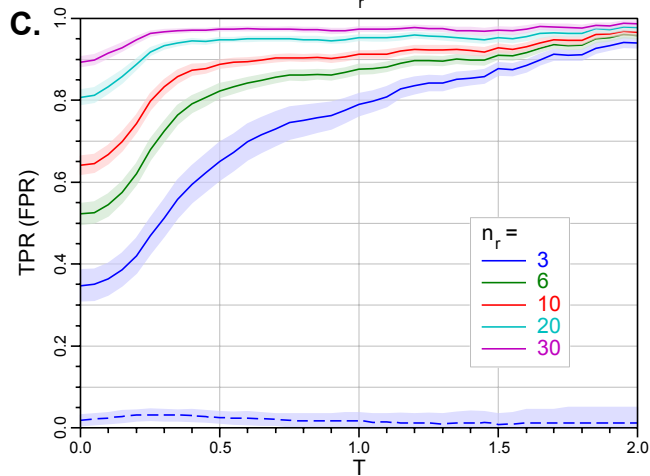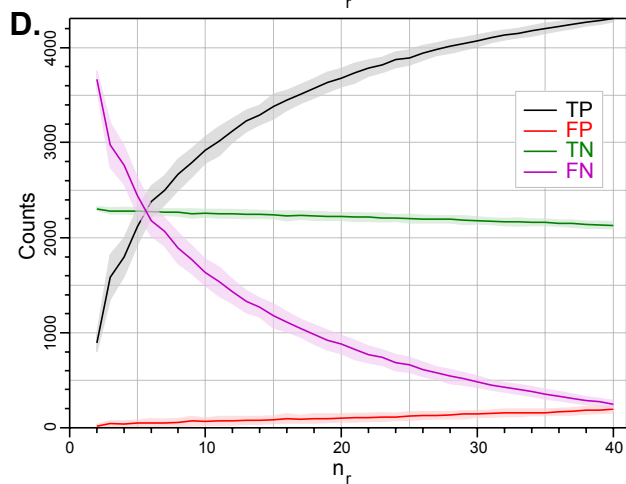

Supplement: Supplemental Material [file supp_053959.115_Supp_FigureS12.pdf]
