# Supplementary material for: How many biological replicates are needed in an RNA-seq experiment and which differential expression tool should you use?
Source: RNA. 2016 Jun;22(6):839–51. doi: 10.1261/rna.053959.115 (PMC4878611; doi:10.1261/rna.053959.115)
Supplement: Supplemental Material [file supp_053959.115_Supp_Figure_Legends.docx]

**Supplementary Figure legends**

**Figure S1:** An example of the log_10_(BH-corrected p-value) vs ${log}_{2}\left( FC \right)$ output data from the differential expression algorithms in this study. The data shown here are from the tool *edgeR (exact)*, averaged over 100 bootstrap iterations with $n_{r}=3$. *edgeR* outputs fold-changes and p-values for 6,885 (96.6%) of the 7,126 input genes, 1,571 (22.1%) of which have BH-corrected p-values $\leq0.05$ (dashed red line). Points shown in yellow represent data with values outside the axes limits.

**Figure S2:** Statistical properties of *BaySeq* as a function of $\left| \log_{2} FC \right|$ threshold, T and the number of replicates, $n_{r}$. As in Figure 2, individual data-points are not shown for clarity; however the points comprising the lines are each an average over 100 bootstrap iterations, with the shaded regions showing the 1-standard-deviation limits. **A:** The fraction of all (7,126) genes called as SDE as a function of the number of replicates (boxplots show the median, quartiles and 95% limits across replicate selections within a bootstrap run). **B:** mean TPR as a function of $n_{r}$ for four fold-change thresholds $T\in\left\{ 0, 0.3, 1, 2 \right\}$ (solid curves, the mean FPR for $T=0$ is shown as the dashed blue curve, for comparison). Data calculated every ${\Delta n}_{r}=1$. **C:** mean TPR as a function of $T$ for $n_{r}\in\left\{ 3, 6, 10, 20, 30 \right\}$ (solid curves, again the mean FPR for $n_{r}=3$ is shown as the dashed blue curve, for comparison). Data calculated every $\Delta T=0.1$ **D:** The number of genes called as TP, FP, TN and FN as a function of $n_{r}$.

**Figure S3:** Statistical properties of *cuffdiff* as a function of $\left| \log_{2} FC \right|$ threshold, T and the number of replicates, $n_{r}$. See Figure S2 for detail on each panel.

**Figure S4:** Statistical properties of *DEGseq* as a function of $\left| \log_{2} FC \right|$ threshold, T and the number of replicates, $n_{r}$. See Figure S2 for detail on each panel.

**Figure S5:** Statistical properties of *DEseq* as a function of $\left| \log_{2} FC \right|$ threshold, T and the number of replicates, $n_{r}$. See Figure S2 for detail on each panel.

**Figure S6:** Statistical properties of *DEseq2* as a function of $\left| \log_{2} FC \right|$ threshold, T and the number of replicates, $n_{r}$. See Figure S2 for detail on each panel.

**Figure S7:** Statistical properties of *EBSeq* as a function of $\left| \log_{2} FC \right|$ threshold, T and the number of replicates, $n_{r}$. See Figure S2 for detail on each panel.

**Figure S8:** Statistical properties of *edgeR (GLM)* as a function of $\left| \log_{2} FC \right|$ threshold, T and the number of replicates, $n_{r}$. See Figure S2 for detail on each panel.

**Figure S9:** Statistical properties of *limma* as a function of $\left| \log_{2} FC \right|$ threshold, T and the number of replicates, $n_{r}$. See Figure S2 for detail on each panel.

**Figure S10:** Statistical properties of *NOISeq* as a function of $\left| \log_{2} FC \right|$ threshold, T and the number of replicates, $n_{r}$. See Figure S2 for detail on each panel.

**Figure S11:** Statistical properties of *PoissonSeq* as a function of $\left| \log_{2} FC \right|$ threshold, T and the number of replicates, $n_{r}$. See Figure S2 for detail on each panel.

**Figure S12:** Statistical properties of *SAMseq* as a function of $\left| \log_{2} FC \right|$ threshold, T and the number of replicates, $n_{r}$. See Figure S2 for detail on each panel.
